# A Comparison of the Brain Parameters of Thais with Normal Cognition, Mild Cognitive Impairment, and Dementia

**DOI:** 10.3390/brainsci15020105

**Published:** 2025-01-23

**Authors:** Pariwat Wisetwongsa, Sitha Piyaselakul, Yudthaphon Vichianin, Pipat Chiewvit, Chatchawan Rattanabannakit, Saowalak Hunnangkul, Natthamon Wongkom, Pathitta Dujada, Vorapun Senanarong

**Affiliations:** 1 Department of Anatomy, Faculty of Medicine, Siriraj Hospital, Mahidol University, Bangkok 10700, Thailand; md.dr.pariwat@gmail.com (P.W.); sithapiya@gmail.com (S.P.); 2 Department of Radiological Technology, Faculty of Medical Technology, Mahidol University, Bangkok 10700, Thailand; 3 Division of Diagnostic Radiology, Department of Radiology, Faculty of Medicine, Siriraj Hospital, Mahidol University, Bangkok 10700, Thailand; pipat8999@gmail.com; 4 Division of Neurology, Department of Internal Medicine, Faculty of Medicine, Siriraj Hospital, Mahidol University, Bangkok 10700, Thailand; chatchawan.rat@mahidol.ac.th (C.R.); natthamon.won@mahidol.ac.th (N.W.); pathitta.duj@mahidol.ac.th (P.D.); 5 Clinical Epidemiology Unit, Faculty of Medicine, Siriraj Hospital, Mahidol University, Bangkok 10700, Thailand; s_hunnangkul@hotmail.com

**Keywords:** brain volume, cortical thickness, dementia, mild cognitive impairment, magnetic resonance imaging

## Abstract

Objectives: This study examined the differences in brain volume and cortical thickness among individuals with normal cognition (NC) and those with NCDs, including mild cognitive impairment (MCI) and dementia. The aim was to identify the brain parameters supporting clinical decision-making for NCDs. Method: A total of 116 participants were categorized into dementia, MCI, and NC groups, and their brain scans using structural magnetic resonance imaging (MRI) were processed and automatedly analyzed with FreeSurfer to obtain the absolute brain volume, volume normalized by intracranial volume (ICV), and cortical thickness. Patients with dementia exhibited a significantly smaller brain volume and cortical thickness than the MCI and NC groups. Results: The left amygdala/ICV ratio demonstrated excellent performance in diagnosing early NCDs, with a cutpoint of ≤0.089, providing 83.30% sensitivity, 84.20% specificity, and 83.82% accuracy. For MCI, a cutpoint of ≤0.099 for the left amygdala/ICV yielded 96.70% sensitivity, 83.30% specificity, and 88.46% accuracy. Conclusions: The findings suggested that reductions in brain volume and cortical thickness correlate with cognitive decline. Utilizing FreeSurfer and MRI data, particularly the left amygdala/ICV ratio, may serve as a valuable biomarker for the early identification of individuals at risk for developing NCDs.

## 1. Introduction

Mild cognitive disorders or MCI and major cognitive disorders or dementia are major parts of neurocognitive disorders (NCDs) [1,2]. Both are spectrum disorders of chronic and progressive cognitive decline. MCI is a mild form of dementia with preserved daily functions. All such NCDs are common in old adults, with their prevalence increasing with advancing age. Dementia is a disease of concern in geriatrics. The World Health Organization has projected that dementia patients will increase from 57.4 million cases globally in 2019 to 152.8 million cases in 2050 [3]. Dementia is one of the most feared health conditions among aging people [4]. Dementia is a significant concern, disrupting daily life and imposing burdens on caregivers, public healthcare systems, and society.

There is currently no curative or reversible treatment for the common types of dementia, such as Alzheimer’s disease, dementia with Lewy bodies, frontotemporal dementia, and vascular dementia [5]. Two monoclonal antibodies, Aducanumab and Lecanemab, approved for beta-amyloid plaque removal, can slow Alzheimer’s progression, with doubts about their effectiveness and adverse reactions [6,7].

Recent research focuses on early NCD stages, particularly the preclinical and MCI phases preceding dementia [8]. The critical role of early detection for NCDs allows interventions, such as cognitive training and lifestyle modification, to prevent or slow disease progression and improve quality of life [9]. Biomarkers for early detection, such as brain volumetric and cortical thickness changes from structural MRI, offer insights into NCDs [10]. Two promising biomarkers [11] for use in clinical settings are brain volumetric and cortical thickness changes obtained from structural MRI.

The detailed information from magnetic resonance (MR) images aids physicians in diagnosis, prognosis prediction, and treatment planning. MRI brain scans are extensively conducted in both clinical and research settings, revealing the extent and nature of anatomical and pathological changes. Specialized software, like FreeSurfer, version 6.0, can automatically process MR images to generate quantitative data, such as brain volume and cortical thickness, with high accuracy [12,13,14]. There are common methods for brain volume analysis, including absolute and intracranial-corrected volumes [15].

Brain volume and cortical thickness naturally decrease with aging [16]. Individuals with NCDs exhibit more pronounced reductions in brain volume and cortical thickness compared to healthy peers [17]. Changes in brain volume and cortical thickness closely align with the progression of clinical symptoms in NCDs [18,19,20]. The severity of these reductions serves as an indicator of the likelihood of impending progression from MCI to dementia [21] and aids in identifying the underlying etiology responsible for the clinical deficit.

There is a study to develop models for temporal changes in NCD biomarkers. It elucidates the correlation between biomarker changes and the progression of Alzheimer’s dementia over time [22]. This model demonstrates that alterations in brain volume and cortical thinning commence many years prior to a typical clinical diagnosis. The application of this model extends to all types of dementia that share similar biomarker changes, encompassing phenomena like brain volume loss and cortical thinning over time.

This study had two objectives: Firstly, to identify a specific brain region and its associated cut-off values that could be used as a clinically relevant biomarker for distinguishing between NC, MCI, and dementia. This has direct implications for clinical practice. Secondly, to explore the relationship between brain parameters (absolute brain volume, ICV-corrected brain volume, and cortical thickness) and diagnostic groups (dementia, MCI, and NC) using linear regression analysis. A key novelty of this research lies in the utilization of a unique dataset comprising a diverse age range within the Thai population. The identified biomarker, characterized by a specific cut-off value, presents a novel application for use within the Thai population. The contributions of this study are significant by (1) providing a comprehensive characterization of the brain structural alterations associated with the progression of NCDs, (2) identifying potential neuroimaging biomarkers that may aid in the early detection and diagnosis of individuals at risk for developing NCDs, and (3) advancing our understanding of the neuroanatomical underpinnings of cognitive decline. It is hypothesized that the findings of this study will facilitate the early detection of NCDs, particularly in the preclinical and MCI stages. This emphasis on early detection is crucial, as early intervention has the potential to significantly decelerate disease progression and enhance patient outcomes.

## 2. Methods

### 2.1. Study Population

Participants were recruited from the Mobility, Cognition, Biomarkers and Artificial Intelligence from Clinical Translation in Dementia Spectrum study (MCAD) at the Neuro-Computational Intelligence for Neurocognitive Disorder Laboratory and Memory Clinic, Faculty of Medicine Siriraj Hospital, Mahidol University, Thailand.

One hundred and twenty participants were recruited. The inclusion criteria were participants aged from 35 to 90 years and having undergone an MRI brain scan (dementia protocol) between January 2019 and February 2022. The exclusion criteria were as follows:

- A serious neurological condition, uncorrected visual or hearing impairment, or a severe psychiatric disorder (e.g., schizophrenia).
- Severe dementia; a bedridden status; uncooperative behavior; a history of brain surgery that may interrupt the intracranial or brain structure; a history or evidence of a brain mass, such as a tumor; hydrocephalus; a history of intracranial hemorrhage; a contraindication for MRI (such as metallic implantation); and claustrophobia.

Four subjects who met the exclusion criteria were eliminated. That left 116 participants (81 females) for the study. Neurologists and psychologists classified the participants into 3 groups: “dementia” (mild to moderate dementia), “MCI”, and “NC”. The diagnoses of dementia and MCI were based on the criteria presented in the *Diagnostic and Statistical Manual of Mental Disorders, Fifth Edition* [23].

Among the participants with dementia, 60.0% had Alzheimer’s disease, 13.3% had frontotemporal dementia, 10% had vascular dementia, 6.7% had Alzheimer’s disease with cerebrovascular disease, and 3.3% each had Lewy body dementia, mixed dementia, and corticobasal degeneration. MCI patients scored >24 on the Thai mental state examination (TMSE), with their cognitive deficits not impeding daily activities. NC participants exhibited no subjective cognitive complaints, normal cognitive performance, and independence functions. All 116 participants underwent laboratory tests to exclude other causes of cognitive decline.

Written informed consent was obtained from all participants. The study protocol received approval from the Human Research Protection Unit, Faculty of Medicine Siriraj Hospital, Mahidol University, before commencement. The approval code is Si 779/2019, and the approval date is 18 November 2019.

### 2.2. Measurements

#### 2.2.1. Baseline Assessments

Clinical data were obtained through physician interviews, encompassing demographic information such as age, sex, body weight, height, exercise frequency, education level, smoking, and alcohol consumption.

The medical data included details of underlying diseases, medications, and cognitive status. The cognitive function assessment for all 116 participants utilized the Thai mental status examination [24], administered by a psychologist. The qualitative evaluation of brain MR images involved a neurologist who assessed the Fazekas scale for white matter lesions, medial temporal lobe atrophy (MTA) score, and the presence of lacunar infarction.

#### 2.2.2. MRI Acquisition and Processing

- MRI technique

All participants underwent a 3.0 T MRI brain scan (Philips Ingenia 3.0, manufactured by Philips, Amsterdam, The Netherlands), with a dementia protocol. The settings of MRI included high-resolution 3D structural T1-weighted (T1W) images and a 3D MP-RAGE sequence with a 1.0 mm isotropic resolution (TR 8.1, TE 3.7, 8 degrees flip angle, and FOV 240 mm (240 × 240 matrix). The MRI scans were administered between 2019 and 2022.

- Morphometric brain volume and cortical thickness assessments

The T1W MR images, initially in Digital Imaging and Communications in Medicine (DICOM) format, were converted to the Neuroimaging Informatics Technology Initiative (NIfTI) format using MRIcron software, version 2.1.63. These images were then analyzed using FreeSurfer software, version 6.0. The FreeSurfer analysis involved motion correction, affine transformation, normalization into Talairach space with image intensity inhomogeneity correction, automatic skull stripping, and segmentation using an image intensity histogram and the FreeSurfer atlas [25]. Segmented brain volume and cortical thickness were obtained for statistical analysis in this study. The analysis focused on selected cortical and subcortical regions, providing parameters such as absolute brain volume, ICV-corrected brain volume, and cortical thickness for further statistical analysis.

### 2.3. Statistical Analysis

The data were analyzed using IBM SPSS Statistics for Windows, version 22. The descriptive statistics for baseline characteristics included frequencies and percentages for the categorical variables and means with standard deviations (SD) or medians with interquartile ranges (IQR) for continuous variables. Comparisons between the NC group, MCI group, and dementia group involved one-way ANOVA for normally distributed data or the Kruskal–Wallis test for abnormal distributions with a post hoc Bonferroni adjustment.

Clinical characteristics, medical data, and brain parameters were subjected to statistical comparisons. The 95% confidence interval of the prevalence was determined using one-way ANOVA and the Kruskal–Wallis test. Probability values (P) less than 0.05 were deemed statistically significant. Linear regression analysis, adjusted for age, education level, and ICV, was employed to ascertain associations between groups and morphometric brain volume (absolute and normalized with the ICV method) and cortical thickness.

A receiver operating characteristic (ROC) curve analysis determined the optimal cutpoint, sensitivity, and specificity for a biomarker in differentiating between groups, while the MedCal software (version 20) generated a positive likelihood ratio, negative likelihood ratio, positive predictive value (PPV), negative predictive value (NPV), and accuracy.

## 3. Results

### 3.1. Participant Recruitment and Baseline Characteristics

**Figure 1:**
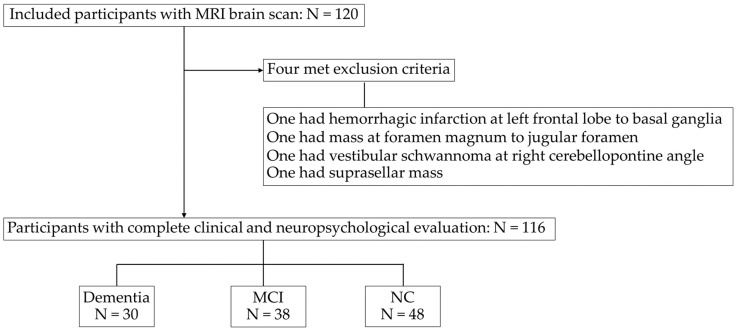
Flowchart of the subjects included in the study. MRI, magnetic resonance imaging. MCI, mild cognitive impairment; NC, normal cognition.

Figure 1 illustrates the flowchart of the studied subject. Of the initial 120 participants, four were excluded due to old hemorrhagic infarctions and intracranial masses. The remaining 116 participants underwent clinical and neuropsychological evaluations. The breakdown of diagnoses was 30 participants (25.86%) with dementia, 38 participants (32.76%) with MCI, and 48 participants (41.38%) with NC. As shown in Table 1, which describes the subject’s characteristics, the participant ages ranged from 39 to 88 years (mean, 65.27 ± 10.56 years), with dementia participants being the oldest, followed by MCI and NC. The majority were female (69.8%), with a mean body mass index of 24.16 ± 4.12 kg/m^2^ (overweight) and mean systolic blood pressure of 130.90 ± 17.55 mmHg (pre-hypertension).

The proportion of individuals with a formal education level over 12 years was higher in the NC group (87.5%) compared to the MCI (65.8%) and dementia (40%) groups.

Regarding underlying diseases, 31.9% of the participants had hypertension, with a higher prevalence in individuals with MCI (50%) compared to dementia (40%) and NC (12.5%). Additionally, 6.9% had psychotic or mood disorders; these were present significantly more often in the dementia group (16.7%) than in the MCI (5.3%) and NC groups (2.1%). Hypnotic sleeping pills were used by 19% of all participants, antidepressant medications by 15.5%, and antipsychotic drugs by 6%. Thyroid hormone replacement therapy was being administered to 6% of all participants. Significant differences were found between the groups for age, education level, TMSE score, hypertension, psychotic and mood disorders, and the use of hypnotic sleeping pills, antidepressants, and antipsychotic medications.

The brain MR images underwent assessment for MTA and white matter lesions by a neurologist. The MTA score [26], indicating Alzheimer’s dementia, and white matter lesions, including the Fazekas scale [27] and lacunar infarction [28], were considered.

An MTA score of 2 or more was significantly more prevalent in dementia (80% for the right, 70% for the left) than in MCI (18.4% for the right, 21.1% for the left) and NC (4.2% for the right, 10.4% for the left). White matter lesions with a Fazekas scale of 2 or more were significantly more common in dementia (50%) than in MCI (18.4%) and NC (8.3%), while lacunar infarction was significantly more frequent in dementia (40%) than in MCI (31.6%) and NC (8.3%).

The high prevalence of white matter lesions in individuals with NCDs reflects the increased occurrence of cerebral small vessel disease reported in Thais and other Asians [29]. It is important to note that a high degree of cerebral small vessel disease can contribute to reductions in brain morphology.

### 3.2. Brain Volume and Cortical Thickness

The brain parameters from the MR images were processed by FreeSurfer through automated segmentation to derive quantitative measurements, including the brain volume, ICV-corrected brain volume (calculated manually by the segmental brain volume multiplied by 100 and divided by the ICV), and cortical thickness. Table 2 shows the regions of interest (ROIs) as parameters for this study, comprising 53 regions for brain volume analysis, 52 regions for brain volume/ICV analysis, and 21 regions for cortical thickness analysis.

Table 3, Table 4 and Table 5 summarize the averages of the brain volume, brain volume normalized by ICV, and cortical thickness in the selected parameters from the ROIs. Additional details are provided in Supplementary Table S1.1–S1.3.

The average total brain volume was 904.98 mL for the dementia group, 960.25 mL for the MCI group, and 1007.12 mL for the NC group. Significant volume differences existed in all pairwise group comparisons for the total cerebral cortex, total cerebral gray matter, subcortical gray matter, total temporal cortex, left and total hippocampus, and left, right, and total amygdala.

All groups exhibited highly significant differences in the volumes of subcortical gray matter, right amygdala, and total amygdala (*p* < 0.01). The total brain volume was 64%, 70%, and 80% of the size of the ICV in the dementia, MCI, and NC groups, respectively (Table 3).

The groups exhibited significant differences in almost all parameters of segmental brain volume corrected with the ICV (Table 4). The parameters that did not show significant differences included the total ventricle/ICV, total cerebral white matter/ICV, total occipital/ICV, total entorhinal cortex/ICV, right hippocampus/ICV, and total hippocampus/ICV.

Regarding the cortical thickness (Table 5), only the right temporal cortex showed a significant difference among all of the compared groups. Except for the right insular cortex, there were no thickness differences between the groups, and the thicknesses of the remaining parameters differed between dementia and NC.

### 3.3. ROC Curve Analysis

ROC curve analyses were conducted for four groups, including a fourth group representing nondementia (combining the MCI and NC data). Table 6, Table 7, Table 8 and Table 9 present the cut-off values of brain parameters derived from ROC analysis to discriminate between the following groups: Table 6 for dementia and non-dementia; Table 7 for dementia and NC; Table 8 for dementia and MCI; and Table 9 for MCI and NC.

The first five parameters with the highest area under the curve (AUC) are listed in the tables below, while the ROC curve analyses of the other brain parameters that provide an AUC ≥ 0.8 are provided in Supplementary Table S2.1–S2.4.

**Figure 2:**
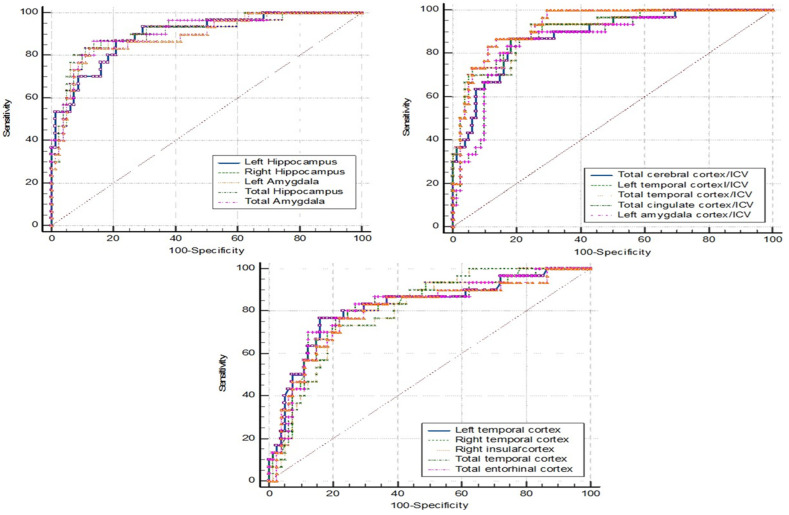
ROC curve analysis: dementia vs. nondementia.

Figure 2 shows the ROC curve analysis of dementia (N = 30) vs. nondementia (N= 86). More details are provided in Table 6, where a cutpoint of ≤0.092 for the left amygdala/ICV ratio demonstrated the best performance. The sensitivity was 86.70%, specificity was 87.20%, PPV was 70.27, NPV was 94.94, and accuracy was 87.07%.

**Figure 3:**
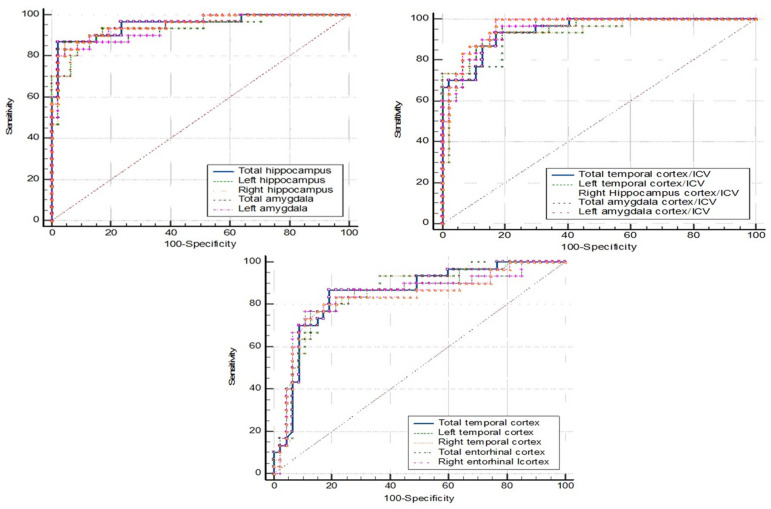
ROC curve analysis: dementia vs. NC.

Figure 3 shows the ROC curve analysis for dementia (N = 30) vs. NC (N = 48). The left amygdala and the left amygdala/ICV gave the same highest accuracy (88.46%). The left amygdala, with a cutpoint of ≤1.349 mL, gave a sensitivity of 90.00%, specificity of 87.50%, PPV of 81.82, and NPV of 93.33. The left amygdala/ICV, with a cutpoint of ≤0.099, showed a sensitivity and specificity at 96.70% and 83.30%, respectively. The corresponding PPV was 78.38 and the NPV was 97.56 (Table 7).

**Figure 4:**
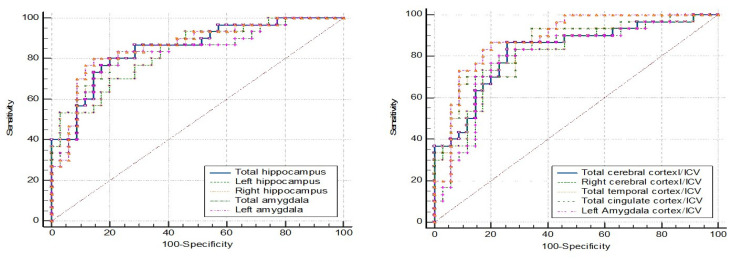
ROC curve analysis: dementia vs. MCI.

The comparison of the ROC curve between dementia (N = 30) and MCI (N = 38) is shown in Figure 4. The left amygdala and the left amygdala/ICV also had the same highest accuracy at 83.82%. The left amygdala, with a cutpoint of ≤1.181 mL, gave a sensitivity of 80.00%, specificity of 86.80%, PPV of 82.76, and NPV of 84.62. The left amygdala/ICV, with a cutpoint of ≤0.089, showed a sensitivity of 83.30%, specificity of 84.20%, PPV of 80.65, and NPV of 86.49 (Table 8).

**Figure 5:**
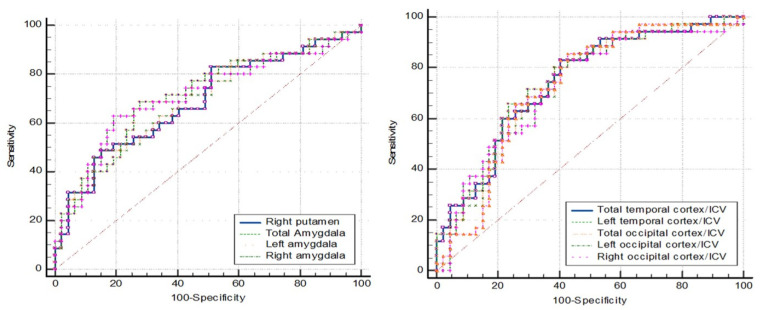
ROC curve analysis: MCI vs. NC.

The ROC curve analysis in Figure 5 represents the MCI (N = 38) vs. NC (N = 48). The ROC curve analysis showed no AUC above 0.8. The AUC below 0.8 lacked the sensitivity and specificity needed for practical application (Table 9).

**Figure 6:**
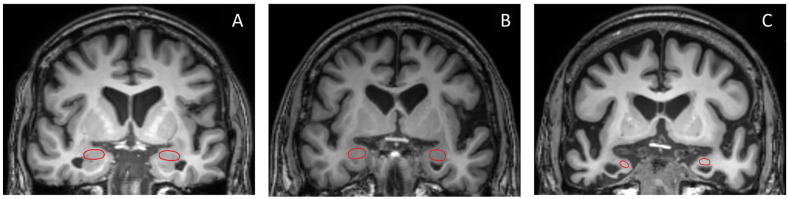
Coronal T1W brain MR images of (**A**) NC, (**B**) MCI, and (**C**) dementia subjects are presented in this study. The red circle highlights the amygdala.

Overall, the ROC curve analysis identified the left amygdala/ICV ratio as the parameter demonstrating the most significant alterations across all compared groups, yielding high sensitivity and specificity for diagnosis. Figure 6 displays the coronal section pictures from T1W MRI of (A) NC, (B) MCI, and (C) dementia subjects in the study. The red circles represent the amygdala, which exhibits prominent size differences between all studied groups [30]. However, in real-world clinical practice, it can be quite challenging for radiologists to visually assess and quantify the degree of amygdala shrinkage.

### 3.4. Linear Regression Analysis

To quantify the difference between brain parameters and the relationship between groups, a linear regression analysis was conducted. The results of the linear regression analyses of the absolute brain volume, brain volume/ICV, and cortical thickness for pairwise group comparisons are detailed in Supplementary Table S3. Only the parameters that were likely to be effective in clinical use and were statistically significant were selected.

**Figure 7:**
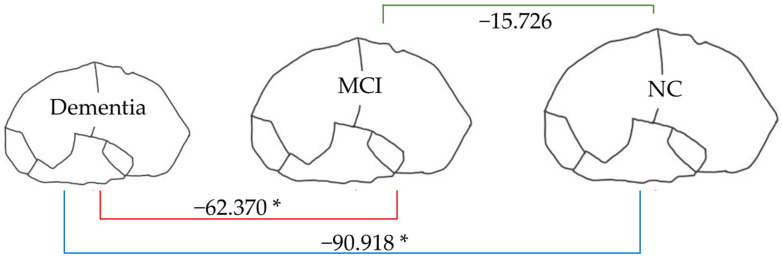
Linear regression analysis of total brain volume. * *p* < 0.05.

**Figure 8:**
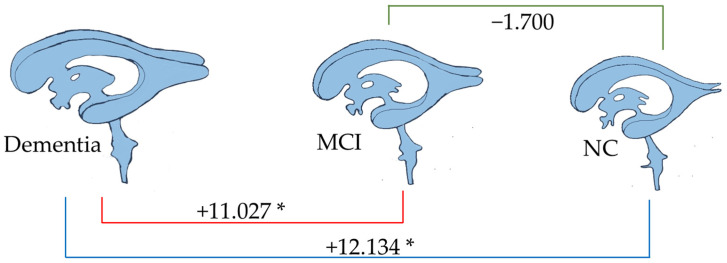
Linear regression analysis of total ventricle volume. * *p* < 0.05.

**Figure 9:**
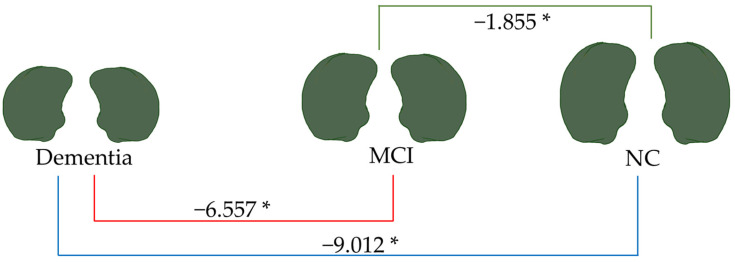
Linear regression analysis of total amygdala volume. * *p* < 0.05.

**Figure 10:**
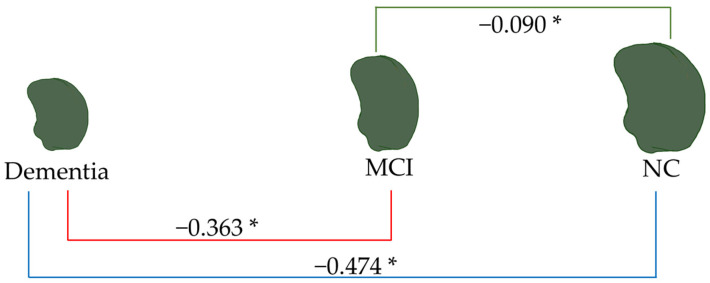
Linear regression analysis of the left amygdala volume. * *p* < 0.05.

Figure 7, Figure 8, Figure 9 and Figure 10 illustrate the volumetric differences (mL) between comparison groups using linear regression analysis. The red, blue, and green lines represent the linear regression analysis results for dementia vs. MCI, dementia vs. NC, and MCI vs. NC, respectively. A star (*) denotes the statistically significant differences. The number on each colored line represents the regression coefficient. A negative (−) sign preceding the number indicates a negative relationship between the comparison groups, signifying that the first group has a lower volume of that brain structure than the second compared group. Conversely, a positive (+) sign indicates a positive relationship, signifying that the first group has a greater volume of that brain structure than the second compared group.

From Figure 7, the total brain volume in dementia (904.976 mL) was lower than that in MCI and NC (62.370 and 90.918 mL, respectively). In contrast, as Figure 8 shows, the total ventricle volume in dementia (43.281 mL) was greater than in MCI and NC (11.027 and 12.314 mL, respectively). These patterns signified changes in the brain structure: the smaller the volume of brain tissue, the larger the size of the brain’s ventricles.

As depicted in Figure 9, the total amygdala volume in the dementia group (2.188 mL) was significantly smaller than that in the MCI group (6.557 mL) and the NC group (9.012 mL), respectively. Figure 10 illustrates the volume difference of the left amygdala between the compared groups. The dementia group exhibited a left amygdala volume that was 0.363 mL lower than the MCI group and 0.474 mL lower than the NC group, respectively.

Notably, from all brain parameters, only the total amygdala volume and left amygdala volume showed significant differences between MCI and NC, with MCI having a 1.855 mL smaller total amygdala volume (*p* < 0.001) and a 0.090 mL smaller left amygdala volume (*p* = 0.044).

## 4. Discussion

In a previous study [22], continuous reductions in brain volume and cortical thickness were observed in dementia and MCI over time. While MRI brain volumetry is recognized as a potential biomarker for clinical settings, there is a lack of studies in Thailand promoting these measures as biomarkers in clinical practice; most investigations were limited to research environments. This study is the first to utilize the quantitative biomarkers of brain volume and cortical thickness changes in diverse Thai cohorts with various dementia subtypes, aiming to contribute to clinical decision-making.

The individuals aged 35 to 90 with dementia, MCI, and NC were observed in our investigation. There are greater neurodegenerative abnormalities in dementia compared to MCI and NC in the Thai population. Absolute brain volumes, including the total cerebral cortex, total cerebral gray matter, subcortical gray matter, total temporal cortex, left and total hippocampus, and left, right, and total amygdala, were significantly smaller in dementia than in MCI and NC. These volumes were also significantly smaller in MCI compared to NC. All selected ROIs exhibited a significantly smaller absolute brain volume in dementia than in NC, confirming our hypothesis of lower absolute brain volume in dementia across all selected ROIs. Additionally, the absolute brain volumes in most selected ROIs in dementia were smaller than those in MCI, while some selected ROIs in MCI had smaller volumes than those in NC.

Normalization of the segmental brain volume by ICV revealed improved significant differences between compared groups. In dementia, all ROIs of the normalized segmental brain volumes were significantly smaller than in NC. Moreover, almost all ROIs of the normalized segmental brain volumes were significantly smaller in dementia than in MCI, except for the total cerebral white matter/ICV and total cerebral cortex/ICV. In MCI, the ROIs of the normalized segmental brain volumes were significantly smaller than in NC, except for the total entorhinal cortex, total hippocampus, and right hippocampus.

Regarding the cortical thickness, all ROIs were thinner in dementia than in NC, and in dementia, they were thinner than in MCI, except for the total, left, and right frontal cortex and the total, left, and right cingulate cortex. The right temporal cortex was the only region significantly thinner in MCI than in NC. Overall, dementia exhibited significantly smaller absolute brain volume, brain volume normalized with ICV, and thinner cortical thickness than both MCI and NC. Additionally, MCI demonstrated smaller brain volume and thinner cortical thickness than NC in specific ROIs.

**Figure 11:**
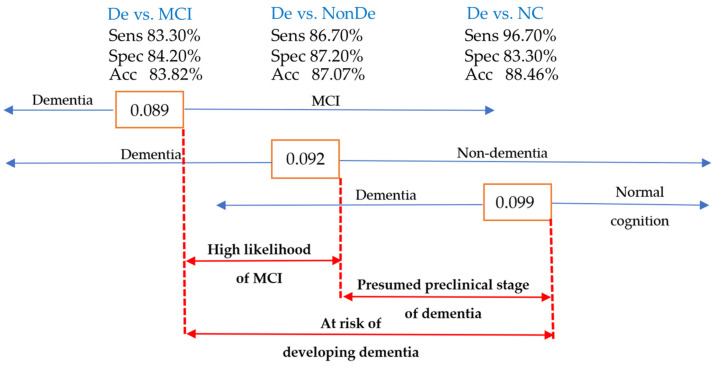
The cutpoint diagram of the left amygdala/ICV as a guide for clinical decision-making in NCD cases. Acc, accuracy; Sens, sensitivity; Spec, specificity; De, dementia; NonDe, nondementia.

The ROC curve analysis identified the left amygdala/ICV with optimal sensitivity and specificity for distinguishing dementia from nondementia, MCI, and NC. Cutpoints of 0.092, 0.089, and 0.099 were proposed for clinical use. Values ≤ 0.089 suggested suspected dementia, those between 0.089 and 0.092 indicated MCI, while values between 0.092 and 0.099 suggested the preclinical stage of dementia. Values > 0.099 indicated normal cognitive function. Individuals with values between 0.089 and 0.099 were considered at risk and should undergo further investigation and management to prevent NCD development. Figure 11 summarizes the proposed cutpoint for the left amygdala/ICV in clinical decision-making for NCDs.

Tau deposition in brain tissue is a common pathology in Alzheimer’s dementia, and its association with reduced amygdala volume has been linked to worsened overall global cognition in individuals in the preclinical stage of the disease or those at risk of developing dementia [30]. Furthermore, MRI-documented atrophy of the hippocampus and amygdala in cognitively intact older adults has been shown to predict dementia over a 6-year follow-up period [31]. These findings highlight amygdala volume loss as a risk factor for dementia development, representing one of the early structural changes in NCDs [32,33,34].

The amygdala, almond-shaped structures within the medial temporal lobe, are subcortical gray structures positioned in front of the hippocampus and are functionally connected together, playing a role in cognition. However, the complex shape and location of the amygdala pose challenges for clinicians to visualize and interpret alterations from MR images [35]. Unlike the hippocampus, the amygdala lacks a clear border, making it difficult to identify volume reduction and compare it with nearby structures.

In contrast, the hippocampus’s surface is easily visualized and evaluated for any shrinkage. Automated segmental brain volume calculations become crucial for accurately determining amygdala volume. While several studies have associated reduced amygdala volume with cognitive decline, fewer have delved into the reduction of amygdala nuclei. A subregion segmentation of amygdala nuclei could provide detailed insights into the specific nuclei responsible for cognitive decline. Additional research into the amygdala and cognitive deterioration in Thai populations, particularly utilizing nuclei volumetric techniques, is proposed.

Linear regression was used in this study with pairwise comparisons for four groups: dementia vs. nondementia, dementia vs. NC, dementia vs. MCI, and MCI vs. NC. Dementia had a total brain volume that was 63.316 mL smaller than that for nondementia, 90.918 mL lower than that for NC, and 62.370 mL less than that for MCI. The total brain volumes of MCI and NC did not differ significantly. Only the total amygdala and the left amygdala volumes demonstrated significant differences between all compared groups. Consequently, the total amygdala and left amygdala volumes had negative linear relationships with the different stages of NCDs.

This study has notable strengths. It is one of the first to investigate the associations between brain volume, cortical thickness, dementia, MCI, and NC in Thais across an extended age range. The inclusion of various dementia types aligns with the clinical setting, enhancing the tool’s relevance for early detection of NCDs. This study introduces cutpoints for evaluating and categorizing patients, proposing brain volume as a potential biomarker for detecting early stages of NCDs. Early identification and intervention can significantly impact patient outcomes. While this study encourages the application of these cutpoints for Thais in clinical and research settings, it acknowledges the need for further trials with diverse age groups and specific diagnoses to validate the proposed values.

There are several clinical implications of this study. Firstly, the identification of the left amygdala/ICV ratio as a sensitive and specific biomarker for NCD diagnosis has significant clinical utility. This biomarker could potentially aid clinicians in early identification of individuals at risk for developing NCDs. Early detection allows for timely interventions, such as cognitive training and lifestyle modifications, which may slow disease progression and improve quality of life. Secondly, this biomarker can also improve diagnostic accuracy. Using the left amygdala/ICV ratio can assist in differentiating between NC, MCI, and dementia, leading to more accurate and confident diagnoses. Thirdly, this biomarker can help stratify individuals based on their risk of progression from MCI to dementia in Thais.

This study has some limitations. Firstly, the sample size, while relatively large, may still be considered moderate. Secondly, the diverse subtypes of dementia with unique pathophysiologies affecting different brain regions may have reduced the study’s statistical power. To address this, focusing on a specific subtype of MCI or dementia could enhance the study’s power. Lastly, the cross-sectional design of this study limits the ability to draw definitive conclusions about the temporal relationship between brain changes and cognitive decline. Further longitudinal studies are needed to confirm these findings and investigate the predictive value of the left amygdala/ICV ratio in predicting future cognitive decline, offering insights into preventative treatments.

Future research should be expanded to include larger and more diverse populations to enhance the generalizability of these findings. The application of various machine learning techniques as alternative presentations of statistics offers the potential to significantly enhance diagnostic accuracy by analyzing a broader range of data. A crucial area for future research involves identifying machine learning models that can accurately predict the probability and rate of conversion from NC to any stage of NCDs. This can be achieved by utilizing the left amygdala/ICV ratio, alone or in combination with other biomarkers, within longitudinal datasets. Such predictive models have the potential to revolutionize early diagnosis and facilitate the development of effective preventive strategies.

## 5. Conclusions

This study investigated brain structural changes in a Thai population, focusing on individuals with NC, MCI, or dementia. The researchers analyzed participants’ brain volume and cortical thickness using MRI data and identified the left amygdala/ICV ratio as a promising biomarker for differentiating between these groups. Significant reductions in brain volume and cortical thickness were observed in individuals with dementia compared to those with MCI and NC. Linear regression analysis confirmed an inverse relationship between amygdala volume and disease severity. These findings suggest that the left amygdala/ICV ratio may serve as a valuable tool for early detection and diagnosis of NCDs in the Thai population, considering the potential influence of ethnicity and cultural factors on brain structure and disease progression.

## Figures and Tables

**Table 1:** Baseline characteristics of the study subjects.

Variable	Total	Dementia	MCI	NC	*p*-Value
	(N = 116)	(N = 30)	(N = 38)	(N = 48)	
Age	65.27 ± 10.56	72.30 ± 11.43	67.53 ± 6.83	59.08 ± 9.00	0.002 *
Sex: female	81 (69.8%)	19 (63.3%)	25 (65.8%)	37 (77.1%)	0.351
BMI	24.16 ± 4.12	22.62 ± 4.35	25.23 ± 3.30	24.26 ± 4.35	0.285
Systolic blood pressure	130.90 ± 17.55	131.47 ± 18.70	134.47 ± 15.35	127.71 ± 18.21	0.442
Education (years)	16 (9–16)	9 (4–16)	16 (9–16)	16 (16–18)	<0.001 *
Education (>12 years)	79 (68.1%)	12 (40%)	25 (65.8%)	42 (87.5%)	<0.001 *
TMSE	28 (25–30)	21 (16.75–24)	28 (26–29)	29 (28–30)	<0.001 *
Smoking	4 (3.4%)	0 (0%)	1 (2.6%)	3 (6.3%)	0.320
Alcohol drinking	14 (12.1%)	1 (3.3%)	8 (21.1%)	5 (10.4%)	0.075
Exercise					
Never	25 (21.6%)	10 (33.3%)	8 (21.1%)	7 (14.6%)	0.056
Sometime	55 (47.4%)	16 (53.3%)	19 (50%)	20 (41.7%)	
Regular 2–3 times/week	36 (31%)	4 (13.3%)	11 (28.9%)	21 (43.8%)	
Comorbidity					
Hypertension	37 (31.9%)	12 (40%)	19 (50%)	6 (12.5%)	0.001 *
Diabetes Mellitus	13 (11.2%)	4 (13.3%)	6 (15.8%)	3 (6.3%)	0.346
Dyslipidemia	13 (11.2%)	4 (13.3%)	6 (15.8%)	3 (6.3%)	0.346
Coronary heart disease	5 (4.3%)	1 (3.3%)	4 (10.5%)	0 (0%)	0.055
Cerebrovascular disease	4 (3.4%)	3 (10%)	0 (0%)	1 (2.1%)	0.064
Thyroid disease	8 (6.9%)	2 (6.7%)	2 (5.3%)	4 (8.3%)	0.854
Psychotic & Mood disorder	8 (6.9%)	5 (16.7%)	2 (5.3%)	1 (2.1%)	0.042 *
Treatment					
Hypnotic sleeping pill	22 (19%)	9 (30%)	10 (26.3%)	3 (6.3%)	0.013 *
Antidepressants	18 (15.5%)	10 (33.3%)	8 (21.1%)	0 (0%)	<0.001 *
Antipsychotic drug	7 (6%)	7 (23.3%)	0 (0%)	0 (0%)	<0.001 *
Thyroid hormone	7 (6%)	0 (0%)	4 (10.5%)	3 (6.3%)	0.194
Visual rating score					
MTA, right (≥2)	33 (28.2%)	24 (80.0%)	7 (18.4%)	2 (4.2%)	<0.001 *
MTA, left (≥2)	34 (29.3%)	21 (70.0%)	8 (21.1)%	5 (10.4%)	<0.001 *
Fazekas scale (≥2)	26 (22.4%)	15 (50.0%)	7 (18.4%)	4 (8.3%)	0.002 *
Lacunar infarction (present)	28 (24.1%)	12 (40.0%)	12 (31.6%)	4 (8.3%)	0.004 *

Data are presented as means ± standard deviation or median (interquartile range); *p*-values are from a one-way ANOVA, Kruskal–Wallis, chi-square, or Fisher’s exact test. * *p*-value < 0.05; MTA, medial temporal lobe atrophy; TMSE, Thai mental state examination.

**Table 2:** The regions of interest included in this brain study.

Brain Volume		Brain Volume/ICV		Cortical Thickness
T. brain	Lt. insular cortex	T. brain	Lt. insular cortex	T. frontal cortex
T. ventricle	Rt. insular cortex	T. ventricle	Rt. insular cortex	Lt. frontal cortex
T. cerebral cortex	T. entorhinal cortex	T. cerebral cortex	T. entorhinal cortex	Rt. frontal cortex
Lt. cerebral cortex	Lt. entorhinal cortex	Lt. cerebral cortex	Lt. entorhinal cortex	T. parietal cortex
Rt. cerebral cortex	Rt. entorhinal cortex	Rt. cerebral cortex	Rt. entorhinal cortex	Lt. parietal cortex
T. cerebral white matter	T. thalamus	T. cerebral white matter	T. thalamus	Rt. parietal cortex
Lt. cerebral white matter	Lt. thalamus	Lt. cerebral white matter	Lt. thalamus	T. temporal cortex
Rt. cerebral white matter	Rt. thalamus	Rt. cerebral white matter	Rt. thalamus	Lt. temporal cortex
T. cerebral gray matter	T. caudate	T. cerebral gray matter	T. caudate	Rt. temporal cortex
T. Subcortical gray matter	Lt. caudate	T. Subcortical gray matter	Lt. caudate	T. occipital cortex
T. intracranial	Rt. caudate	T. frontal cortex	Rt. caudate	Lt. occipital cortex
T. frontal cortex	T. putamen	Lt. frontal cortex	T. putamen	Rt. occipital cortex
Lt. frontal cortex	Lt. putamen	Rt. frontal cortex	Lt. putamen	T. cingulate cortex
Rt. frontal cortex	Rt. putamen	T. parietal cortex	Rt. putamen	Lt. cingulate cortex
T. parietal cortex	T. pallidum	Lt. parietal cortex	T. pallidum	Rt. cingulate cortex
Lt. parietal cortex	Lt. pallidum	Rt. parietal cortex	Lt. pallidum cortex	T. Insular cortex
Rt. parietal cortex	Rt. pallidum	T. temporal cortex	Rt. pallidum cortex	Lt. insular cortex
T. temporal cortex	T. hippocampus	Lt. temporal cortex	T. hippocampus	Rt. insular cortex
Lt. temporal cortex	Lt. hippocampus	Rt. temporal cortex	Lt. hippocampus	T. entorhinal cortex
Rt. temporal cortex	Rt. hippocampus	T. occipital cortex	Rt. hippocampus	Lt. entorhinal cortex
T. occipital cortex	T. amygdala	Lt. occipital cortex	T. amygdala	Rt. entorhinal cortex
Lt. occipital cortex	Lt. amygdala	Rt. occipital cortex	Lt. amygdala	
Rt. occipital cortex	Rt. amygdala	T. cingulate cortex	Rt. amygdala	
T. cingulate cortex	T. nucleus accumbens	Lt. cingulate cortex	T. nucleus accumbens	
Lt. cingulate cortex	Lt. nucleus accumbens	Rt. cingulate cortex	Lt. nucleus accumbens	
Rt. cingulate cortex	Rt. nucleus accumbens	T. Insular cortex	Rt. nucleus accumbens	
T. insular cortex				

Lt., left; Rt., right; T., total.

**Table 3:** Brain volume.

Brain Volume	Dementia	MCI	NC
Total brain *	904.976 ± 110.745	960.253 ± 97.965	1007.115 ± 86.521
Total ventricle *, **	43.281 ± 21.031	27.110 ± 13.989	21.855 ± 14.992
Total cerebral cortex *, **, ***	347.663 ± 43.955	381.201 ± 39.533	402.531 ± 35.812
Total cerebral white matter *	393.344 ± 59.982	410.422 ± 47.666	428.231 ± 44.670
Total cerebral gray matter *, **, ***	486.572 ± 58.173	523.172 ± 52.833	551.471 ± 44.498
Total subcortical *, **, ***	45.430 ± 4.985	49.188 ± 4.770	52.227 ± 4.006
Total intracranial *	1407.296 ± 224.483	1328.430 ± 233.226	1258.662 ± 229.822
Total frontal cortex *	126.372 ± 16.040	134.444 ± 13.937	140.343 ± 13.168
Total parietal cortex *, **	81.042 ± 11.300	90.357 ± 9.820	95.571 ± 9.583
Total temporal cortex *, **	76.993 ± 13.129	88.817 ± 10.327	94.702 ± 8.833
Total occipital cortex *, ***	37.646 ± 6.002	38.881 ± 5.072	41.922 ± 4.798
Total cingulate cortex *, **	14.774 ± 2.158	16.196 ± 2.143	17.210 ± 1.926
Total entorhinal cortex *, **	2.687 ± 0.829	3.578 ± 0.860	3.757 ± 0.579
Total hippocampus *, **, ***	5.913 ± 0.989	7.345 ± 0.918	7.832 ± 0.634
Left hippocampus *, **, ***	2.947 ± 0.516	3.600 ± 0.427	3.849 ± 0.317
Right hippocampus *, **	2.966 ± 0.530	3.744 ± 0.514	3.984 ± 0.345
Total amygdala *, **, ***	2.188 ± 0.541	2.858 ± 0.450	3.152 ± 0.331
Left amygdala *, **, ***	0.997 ± 0.256	1.360 ± 0.210	1.488 ± 0.162
Right amygdala *, **, ***	1.191 ± 0.307	1.498 ± 0.270	1.663 ± 0.186

Values are reported as means ± SD. * *p* < 0.05 for dementia vs. NC; ** *p* < 0.05 for dementia vs. MCI; *** *p* < 0.05 for MCI vs. NC.

**Table 4:** Brain volume/total intracranial volume.

Brain Volume/ICV	Dementia	MCI	NC
Total brain *, **, ***	64.211 (60.608–68.340)	70.366 (66.306–80.986)	80.487 (71.631–94.783)
Total ventricle *, **	2.918 (2.241–3.301)	1.941 (1.376–2.561)	1.433 (0.973–2.105)
Total cerebral cortex *, **, ***	12.246 (11.368–13.199)	14.299 (13.261–15.779)	16.346 (14.278–18.613)
Total cerebral white matter *, ***	28.438 (25.255–30.511)	30.090 (27.130–35.755)	34.162 (29.596–40.415)
Total cerebral gray matter *, **, ***	34.946 (32.281–36.256)	39.215 (36.395–43.480)	44.800 (39.589–51.232)
Total subcortical *, **, ***	3.278 (2.946–3.490)	3.679 (3.275–4.217)	4.271 (3.731–4.906)
Total frontal cortex *, **, ***	9.082 (8.141–9.550)	9.917 (9.418–11.043)	11.395 (9.979–12.784)
Total parietal cortex *, **, ***	5.810 (5.263–6.216)	6.680 (6.154–7.634)	7.587 (6.691–8.944)
Total temporal cortex *, **, ***	5.367 (4.975–6.239)	6.672 (6.090–7.155)	7.578 (6.719–8.694)
Total occipital cortex *, ***	2.721 (2.361–2.863)	2.807 (2.649–3.171)	3.401 (2.890–3.959)
Total cingulate cortex *, **, ***	1.058 (0.984–1.093)	1.196 (1.106–1.321)	1.380 (1.211–1.601)
Total entorhinal cortex *, **	0.195 (0.136–0.244)	0.279 (0.227–0.317)	0.288 (0.257–0.350)
Total hippocampus *, **, ***	0.419 (0.359–0.480)	0.563 (0.475–0.645)	0.638 (0.544–0.758)
Left hippocampus *, **, ***	0.209 (0.177–0.237)	0.270 (0.236–0.309)	0.310 (0.269–0.372)
Right hippocampus *, **	0.199 (0.175–0.252)	0.291 (0.231–0.334)	0.327 (0.274–0.379)
Total amygdala *, **, ***	0.157 (0.124–0.182)	0.218 (0.186–0.244)	0.253 (0.218–0.303)
Left amygdala *, **, ***	0.071 (0.060–0.084)	0.102 (0.092–0.119)	0.120 (0.103–0.142)
Right amygdala *, **, ***	0.088 (0.066–0.098)	0.113 (0.096–0.133)	0.135 (0.115–0.159)

Values are reported as median (interquartile range). * *p* < 0.05 for dementia vs. NC; ** *p* < 0.05 for dementia vs. MCI; *** *p* < 0.05 for MCI vs. NC.

**Table 5:** Cortical thickness.

Cortical Thickness	Dementia	MCI	NC
Total frontal cortex *	4.692 (4.502–4.843)	4.820 (4.661–4.911)	4.876 (4.670–5.031)
Total parietal cortex *, **	4.122 (3.898–4.296)	4.248 (4.134–4.411)	4.314 (4.151–4.439)
Total temporal cortex *, **	5.059 (4.765–5.267)	5.395 (5.261–5.545)	5.556 (5.419–5.695)
Total occipital cortex *, **	3.687 (3.592–3.794)	3.846 (3.709–3.949)	3.855 (3.735–3.936)
Total cingulate cortex *	4.769 (4.521–4.972)	4.985 (4.771–5.080)	5.053 (4.762–5.225)
Total entorhinal cortex *, **	5.859 (4.485–6.697)	7.116 (6.179–7.471)	7.302 (6.954–7.601)
Total insular cortex *, **	5.150 (4.887–5.666)	5.755 (5.502–6.005)	5.785 (5.592–6.026)

Values are reported as median (interquartile range). * *p* < 0.05 for dementia vs. NC; ** *p* < 0.05 for dementia vs. MCI.

**Table 6:** Cut-offs of brain parameters for discriminate dementia and nondementia groups.

Brain Volume	AUC	*p*-Value	Cut-Off (mL)	Sens	Spec	PLR	NLR	PPV	NPV	ACC
Left hippocampus	0.897	<0.001	3.413	80.00	81.40	4.30	0.25	60.00	92.11	81.03
Right hippocampus	0.903	<0.001	3.542	86.70	83.70	5.32	0.16	65.00	94.74	84.48
Left amygdala	0.912	<0.001	1.280	86.70	81.40	4.66	0.16	61.90	94.59	82.76
Total hippocampus	0.910	<0.001	6.883	86.70	86.00	6.21	0.15	68.42	94.87	86.21
Total amygdala	0.891	<0.001	2.651	83.30	87.20	6.52	0.19	69.44	93.75	86.21
**Brain Volume/ICV**	**AUC**	***p*** **-Value**	**Cut-Off (%)**	**Sens**	**Spec**	**PLR**	**NLR**	**PPV**	**NPV**	**ACC**
Total cerebral cortex/ICV	0.884	<0.001	26.787	86.70	82.56	4.97	0.16	63.41	94.67	83.62
Left temporal cortex/ICV	0.912	<0.001	3.182	86.70	82.60	4.97	0.16	63.41	94.67	83.62
Total temporal cortex/ICV	0.909	<0.001	6.367	86.70	81.40	4.66	0.16	61.9	94.59	82.76
Total cingulate cortex/ICV	0.871	<0.001	1.112	83.30	83.70	5.12	0.20	64.1	93.51	83.62
Left amygdala/ICV	0.933	<0.001	0.092	86.70	87.20	6.78	0.15	70.27	94.94	87.07
**Cortical Thickness**	**AUC**	***p*** **-Value**	**Cut-Off (mm)**	**Sens**	**Spec**	**PLR**	**NLR**	**PPV**	**NPV**	**ACC**
Left temporal cortex	0.817	<0.001	2.655	80.00	75.60	3.28	0.26	53.33	91.55	76.72
Right temporal cortex	0.814	<0.001	2.660	80.00	74.40	3.13	0.27	52.17	91.43	75.86
Right insular cortex	0.806	<0.001	2.695	73.30	80.20	3.71	0.33	56.41	89.61	78.45
Total temporal cortex	0.816	<0.001	5.342	80.00	74.40	3.13	0.27	52.17	91.43	75.86
Total entorhinal cortex	0.807	<0.001	6.870	83.30	72.10	2.88	0.24	51.02	92.54	75.00

ACC, accuracy; AUC, area under the curve; NLR, negative likelihood ratio; NPV, negative predictive value; PLR, positive likelihood ratio; PPV, positive predictive value; Sens, sensitivity; Spec, specificity.

**Table 7:** Cut-offs of brain parameters for discriminate dementia and NC groups.

Brain Volume	AUC	*p*-Value	Cut-Off (mL)	Sens	Spec	PLR	NLR	PPV	NPV	ACC
Total hippocampus	0.953	<0.001	7.209	90.00	85.40	6.17	0.12	79.41	93.18	87.18
Left hippocampus	0.944	<0.001	3.520	93.30	83.30	5.60	0.08	77.78	95.24	87.18
Right hippocampus	0.941	<0.001	3.678	93.30	83.30	5.60	0.08	77.78	95.24	87.18
Total amygdala	0.939	<0.001	2.827	86.70	87.50	6.93	0.15	81.25	91.30	87.18
Left amygdala	0.951	<0.001	1.349	90.00	87.50	7.20	0.11	81.82	93.33	88.46
**Brain Volume/ICV**	**AUC**	***p*** **-Value**	**Cut-Off (%)**	**Sens**	**Spec**	**PLR**	**NLR**	**PPV**	**NPV**	**ACC**
Total temporal cortex	0.945	<0.001	6.514	93.30	83.30	5.60	0.08	77.78	95.24	87.18
Left temporal cortex/ICV	0.951	<0.001	3.264	90.00	85.40	6.17	0.12	79.41	93.18	87.18
Right hippocampus/ICV	0.919	<0.001	0.267	93.30	81.20	4.98	0.08	75.68	95.12	85.90
Total amygdala	0.956	<0.001	0.216	96.70	81.20	5.16	0.04	76.32	97.5	87.18
Left amygdala/ICV	0.964	<0.001	0.099	96.70	83.30	5.80	0.04	78.38	97.56	88.46
**Cortical Thickness**	**AUC**	***p*** **-Value**	**Cut-Off (mm)**	**Sens**	**Spec**	**PLR**	**NLR**	**PPV**	**NPV**	**ACC**
Total temporal cortex	0.847	<0.001	5.409	86.70	79.20	4.16	0.17	72.22	90.48	82.05
Left temporal cortex	0.837	<0.001	2.655	80.00	77.10	3.49	0.26	68.57	86.05	78.21
Right temporal cortex	0.850	<0.001	2.660	80.00	81.20	4.27	0.25	72.73	86.67	80.77
Total entorhinal cortex	0.842	<0.001	6.879	83.30	79.20	4.00	0.21	71.43	88.37	80.77
Right entorhinal cortex	0.832	<0.001	3.383	80.00	83.30	4.80	0.24	75.00	86.96	82.05

ACC, accuracy; AUC, area under the curve; NLR, negative likelihood ratio; NPV, negative predictive value; PLR, positive likelihood ratio; PPV, positive predictive value; Sens, sensitivity; Spec, specificity.

**Table 8:** Cut-offs of brain parameters for discriminate dementia and MCI.

Brain Volume	AUC	*p*-Value	Cut-Off (mL)	Sens	Spec	PLR	NLR	PPV	NPV	ACC
Total hippocampus	0.856	<0.001	6.724	80.00	78.90	3.80	0.25	75.00	83.33	79.41
Left hippocampus	0.838	<0.001	3.363	76.70	71.10	2.65	0.33	67.65	79.41	73.53
Right hippocampus	0.856	<0.001	3.396	80.00	81.60	4.34	0.25	77.42	83.78	80.88
Total amygdala	0.831	<0.001	2.651	83.30	76.30	3.52	0.22	73.53	88.64	82.05
Left amygdala	0.861	<0.001	1.181	80.00	86.80	6.08	0.23	82.76	84.62	83.82
**Brain Volume/ICV**	**AUC**	***p*** **-Value**	**Cut-Off (%)**	**Sens**	**Spec**	**PLR**	**NLR**	**PPV**	**NPV**	**ACC**
Total cerebral cortex/ICV	0.836	<0.001	26.787	86.67	76.32	3.66	0.17	74.29	87.88	80.88
Right cerebral cortex/ICV	0.826	<0.001	13.397	83.30	73.70	3.17	0.23	71.43	84.85	77.94
Total temporal cortex/ICV	0.864	<0.001	6.367	86.70	73.70	3.29	0.18	72.22	87.5	79.41
Total cingulate cortex/ICV	0.818	<0.001	1.105	80.00	78.90	3.80	0.25	75.00	83.33	79.41
Left amygdala/ICV	0.893	<0.001	0.089	83.30	84.20	5.28	0.20	80.65	86.49	83.82

ACC, accuracy; AUC, area under the curve; NLR, negative likelihood ratio; NPV, negative predictive value; PLR, positive likelihood ratio; PPV, positive predictive value; Sens, sensitivity; Spec, specificity.

**Table 9:** Cut-offs of brain parameters for discriminate MCI and NC groups.

Brain Volume	AUC	*p*-Value	Cut-Off (mL)	Sens	Spec	PLR	NLR	PPV	NPV	ACC
Right putamen	0.700	0.002	4.254	65.80	60.40	1.66	0.57	56.82	69.05	62.79
Total amygdala	0.724	<0.001	2.964	71.10	72.90	2.62	0.40	67.5	76.09	72.09
Left amygdala	0.711	0.001	1.462	71.10	60.40	1.80	0.48	58.7	72.5	65.12
Right amygdala	0.717	0.001	1.602	71.10	64.60	2.01	0.45	61.36	73.81	67.44
**Brain Volume/ICV**	**AUC**	***p*** **-Value**	**Cut-Off (%)**	**Sens**	**Spec**	**PLR**	**NLR**	**PPV**	**NPV**	**ACC**
Total temporal cortex/ICV	0.716	0.001	6.990	71.10	62.50	1.89	0.46	60.00	73.17	66.28
Left temporal cortex/ICV	0.723	<0.001	3.564	76.30	60.40	1.93	0.39	60.42	76.32	67.44
Total occipital cortex/ICV	0.715	0.001	3.168	76.30	60.40	1.93	0.39	60.42	76.32	67.44
Left occipital cortex/ICV	0.712	0.001	1.489	73.70	60.40	1.86	0.44	59.57	74.36	66.28
Right occipital cortex/ICV	0.714	0.001	1.674	78.90	60.40	1.99	0.35	61.22	78.38	68.60

ACC, accuracy; AUC, area under the curve; NLR, negative likelihood ratio; NPV, negative predictive value; PLR, positive likelihood ratio; PPV, positive predictive value; Sens, sensitivity; Spec, specificity.

## Data Availability

The data that support the findings of this study are available from the corresponding author upon reasonable request.

## Supplementary Materials

The following supporting information can be downloaded at: https://www.mdpi.com/article/10.3390/brainsci15020105/s1, Table S1.1. Absolute segmental brain volume; Table S1.2. Segmental brain volume/total intracranial volume; Table S1.3. Cortical thickness; Table S2.1. ROC curve analysis: dementia (n = 30) vs. non-dementia (n = 86); Table S2.2. ROC curve analysis: dementia (n = 30) vs. NC (n = 48); Table S2.3. ROC curve analysis: dementia (n = 30) vs. MCI (n = 38); Table S2.4. ROC curve analysis: MCI (n = 38) vs. NC (n = 48); Table S3. Linear regression analysis.
