# Supplementary material for: A Comparison of the Brain Parameters of Thais with Normal Cognition, Mild Cognitive Impairment, and Dementia
Source: Brain Sci. 2025 Jan 23;15(2):105. doi: 10.3390/brainsci15020105 (PMC11852455; doi:10.3390/brainsci15020105)
Supplement: Supplementary file 1 [file brainsci-15-00105-s001.zip › brainsci-3371077-supplementary.pdf]

Supplementary 1

Table S1.1. Absolute segmental brain volume

Segmental brain volumes	Dementia (n=30)		MCI (n=38)		NC (n=48)		p-value
	Means	SD	Means	SD	Means	SD	
Total brain	904.976	110.745	960.253	97.965	1007.115	86.521	<0.001†, 0.064‡ , 0.084§
Total ventricle	43.281	21.031	27.110	13.989	21.855	14.992	<0.001†, <0.001‡ , 0.433§
Total cerebral cortex	347.663	43.955	381.201	39.533	402.531	35.812	<0.001†, 0.002‡ , 0.041§
Left cerebral cortex	174.633	21.344	190.478	19.967	201.153	17.965	<0.001†, 0.004‡ , 0.040§
Right cerebral cortex	173.030	23.317	190.723	19.712	201.377	17.925	<0.001†, 0.001‡ , 0.047§
Total cerebral white matter	393.344	59.982	410.422	47.666	428.231	44.670	<0.010†, 0.493‡ , 0.311§
Left cerebral white matter	197.008	29.333	205.621	24.348	214.034	22.265	<0.012†, 0.479‡ , 0.368§
Right cerebral white matter	196.336	31.268	204.801	23.467	214.197	22.467	<0.009†, 0.521‡ , 0.271§
Total cerebral gray matter	486.572	58.173	523.172	52.833	551.471	44.498	<0.001†, 0.012‡ , 0.036§
Subcortical cerebral gray matter	45.430	4.985	49.188	4.770	52.227	4.006	<0.001†, 0.003‡ , 0.008§
Total intracranium	1407.296	224.483	1328.430	233.226	1258.662	229.822	<0.019†, 0.487‡ , 0.493§
Total frontal cortex	126.372	16.040	134.444	13.937	140.343	13.168	<0.001†, 0.065‡ , 0.175§
Left frontal cortex	63.830	7.888	67.590	7.224	70.698	6.665	<0.001†, 0.102‡ , 0.146§
Right frontal cortex	62.542	8.312	66.853	6.847	69.645	6.615	<0.001†, 0.046‡ , 0.226§
Total parietal cortex	81.042	11.300	90.357	9.820	95.571	9.583	<0.001†, 0.001‡ , 0.058§
Left parietal cortex	40.720	5.759	44.835	5.102	47.276	4.890	<0.001†, 0.005‡ , 0.098§
Right parietal cortex	40.322	5.759	45.522	4.824	48.295	4.815	<0.001†,<0.001‡ , 0.040§
Total temporal cortex	76.993	13.129	88.817	10.327	94.702	8.833	<0.001†, <0.001‡ , 0.035§
Left temporal cortex	39.144	6.431	44.866	5.244	47.967	4.279	<0.001†, <0.001‡ , 0.022§
Right temporal cortex	37.849	7.141	43.951	5.202	46.735	4.653	<0.001†, <0.001‡ , 0.069§
Total occipital cortex	37.646	6.002	38.881	5.072	41.922	4.798	<0.002†, 1.000‡ , 0.025§
Left occipital cortex	18.289	2.912	18.852	2.484	20.215	2.440	<0.005†, 1.000‡ , 0.050§
Right occipital cortex	19.357	3.195	20.029	2.711	21.707	2.532	<0.001†, 0.969‡ , 0.019§
Total cingulate cortex	14.774	2.158	16.196	2.143	17.210	1.926	<0.001†, 0.017‡ , 0.076§
Left cingulate cortex	7.387	0.000	8.057	1.152	8.665	1.115	<0.001†, 0.049‡ , 0.042§
Right cingulate cortex	7.386	1.269	8.139	1.230	8.546	1.074	<0.001†, 0.030‡ , 0.344§
Total insular cortex	11.302	1.623	12.596	1.853	12.673	1.139	<0.001†, 0.002‡ , 1.000§
Left insular cortex	5.692	0.834	6.346	0.951	6.335	0.652	<0.003†, 0.004‡ , 1.000§
Right insular cortex	5.610	0.877	6.251	0.943	6.338	0.563	<0.001†, 0.004‡ , 1.000§
Total entorhinal cortex	2.687	0.829	3.578	0.860	3.757	0.579	<0.001†, <0.001‡ , 0.815§
Left entorhinal cortex	1.407	0.453	1.832	0.483	1.895	0.327	<0.001†, <0.001‡ , 1.000§
Right entorhinal cortex	1.280	0.450	1.746	0.459	1.863	0.335	<0.001†, <0.001‡ , 0.580§
Total thalamus	11.555	1.392	11.966	1.286	12.656	1.496	<0.003†, 0.701‡ , 0.076§
Left thalamus	5.975	0.706	6.160	0.787	6.522	0.862	<0.012†, 1.000‡ , 0.118§
Right thalamus	5.580	0.802	5.805	0.572	6.134	0.671	<0.002†, 0.530‡ , 0.083§
Total caudate	5.780	1.110	5.870	0.843	6.235	0.696	<0.078†, 1.000‡ , 0.166§
Left caudate	2.838	0.543	2.895	0.412	3.049	0.359	0.079
Right caudate	2.942	0.628	2.976	0.460	3.186	0.358	<0.086†, 1.000‡ , 0.129§
Total putamen	7.403	0.959	8.054	0.973	8.661	0.826	<0.001†, 0.013‡ , 0.008§
Left putamen	3.708	0.516	4.006	0.502	4.299	0.445	<0.001†, 0.039‡ , 0.018§
Right putamen	3.696	0.540	4.048	0.503	4.362	0.406	<0.001†, 0.009‡ , 0.009§
Total pallidum	3.495	0.424	3.636	0.436	3.737	0.443	0.063
Left pallidum	1.778	0.238	1.851	0.207	1.862	0.243	0.266
Right pallidum	1.717	0.246	1.785	0.252	1.875	0.218	<0.015†, 0.739‡ , 0.249§
Total hippocampus	5.913	0.989	7.345	0.918	7.832	0.634	<0.001†, <0.001‡ , 0.024§
Left hippocampus	2.947	0.516	3.600	0.427	3.849	0.317	<0.001†, <0.001‡ , 0.019§
Right hippocampus	2.966	0.530	3.744	0.514	3.984	0.345	<0.001†, <0.001‡ , 0.052§
Total amygdala	2.188	0.541	2.858	0.450	3.152	0.331	<0.001†, <0.001‡ , 0.004§
Left amygdala	0.997	0.256	1.360	0.210	1.488	0.162	<0.001†, <0.001‡ , 0.014§
Right amygdala	1.191	0.307	1.498	0.270	1.663	0.186	<0.001†, <0.001‡ , 0.009§
Total nucleus accumbens	0.579	0.164	0.743	0.134	0.837	0.166	<0.001†, <0.001‡ , 0.019§
Left nucleus accumbens	0.255	0.093	0.335	0.075	0.388	0.089	<0.001†, 0.001‡ , 0.016§
Right nucleus accumbens	0.324	0.080	0.409	0.073	0.450	0.086	<0.001†, <0.001‡ , 0.061§

Data are presented as means and standard deviation (ml). P-value corresponds to one-way independent ANOVA test. Pairwise comparisons of each group † = Dementia vs NC p-value, ‡ = Dementia vs MCI p-value and § = MCI vs NC p-value. MCI, Mild cognitive impairment; NC, Normal control

Table S1.2. Segmental brain volume/total intracranial volume

Segmental brain volume/ intracranial volume	Dementia (n=30)		MCI (n=38)		NC (n=48)		p-value
	Median	IQR	Median	IQR	Median	IQR	
Total brain	64.211	60.608-68.340	70.366	66.306-80.986	80.487	71.631-94.783	<0.001†, <0.001‡, 0.016§
Total ventricle	2.918	2.241-3.301	1.941	1.376-2.561	1.433	0.973-2.105	<0.001†, 0.001‡, 0.221§
Total cerebral cortex	12.246	11.368-13.199	14.299	13.261-15.779	16.346	14.278-18.613	<0.001†, <0.001‡, 0.017§
Left cerebral cortex	12.545	11.465-13.263	14.159	13.367-15.711	16.255	14.339-18.949	<0.001†, <0.001‡, 0.018§
Right cerebral cortex	12.246	11.368-13.199	14.299	13.261-15.779	16.346	14.278-18.613	<0.001†, <0.001‡, 0.015§
Total cerebral white matter	28.438	25.255-30.511	30.090	27.130-35.755	34.162	29.596-40.415	<0.001†, 0.053‡, 0.026§
Left cerebral white matter	14.199	12.732-15.189	15.150	13.593-17.990	16.891	14.876-20.197	<0.001†, 0.039‡, 0.030§
Right cerebral white matter	14.006	12.665-15.228	15.010	13.515-17.765	17.171	14.720-20.199	<0.001†, 0.067‡, 0.028§
Total cerebral gray matter	34.946	32.281-36.256	39.215	36.395-43.480	44.800	39.589-51.232	<0.001†, 0.001‡, 0.011§
Subcortical cerebral gray matter	3.278	2.946-3.490	3.679	3.275-4.217	4.271	3.731-4.906	<0.001†, 0.002‡, 0.016§
Total frontal cortex	9.082	8.141-9.550	9.917	9.418-11.043	11.395	9.979-12.784	<0.001†, 0.002‡, 0.019§
Left frontal cortex	4.562	4.136-4.808	5.009	4.746-5.594	5.727	4.961-6.470	<0.001†, 0.002‡, 0.025§
Right frontal cortex	4.524	4.046-4.825	4.970	4.648-5.571	5.595	5.007-6.382	<0.001†, 0.002‡, 0.022§
Total parietal cortex	5.810	5.263-6.216	6.680	6.154-7.634	7.587	6.691-8.944	<0.001†, <0.001‡, 0.018§
Left parietal cortex	2.937	2.612-3.157	3.336	3.067-3.716	3.841	3.256-4.413	<0.001†, 0.001‡, 0.023§
Right parietal cortex	2.878	2.652-3.088	3.385	3.072-3.825	3.804	3.406-4.525	<0.001†, <0.001‡, 0.015§
Total temporal cortex	5.367	4.975-6.239	6.672	6.090-7.155	7.578	6.719-8.694	<0.001†, <0.001‡, 0.012§
Left temporal cortex	2.826	2.514-3.111	3.378	3.139-3.587	3.861	3.416-4.401	<0.001†, <0.001‡, 0.479§
Right temporal cortex	2.632	2.449-3.109	3.322	3.020-3.582	3.723	3.273-4.275	<0.001†, <0.001‡, 0.024§
Total occipital cortex	2.721	2.361-2.863	2.807	2.649-3.171	3.401	2.890-3.959	<0.001†, 0.156‡, 0.004§
Left occipital cortex	1.321	1.161-1.437	1.364	1.278-1.561	1.646	1.382-1.896	<0.001†, 0.188‡, 0.004§
Right occipital cortex	1.385	1.226-1.494	1.452	1.365-1.658	1.779	1.497-2.038	<0.001†, 0.099‡, 0.005§
Total cingulate cortex	1.058	0.984-1.093	1.196	1.106-1.321	1.380	1.211-1.601	<0.001†, <0.001‡, 0.016§
Left cingulate cortex	0.525	0.490-0.579	0.597	0.530-0.685	0.688	0.610-0.790	<0.001†, 0.003‡, 0.005§
Right cingulate cortex	0.516		0.608	0.560-0.691	0.672	0.573-0.807	<0.001†, 0.001‡, 0.110§
Total Insular cortex	0.817	0.771-0.859	0.885	0.815-1.024	1.022	0.851-1.207	<0.001†, 0.042‡, 0.041§
Left insular cortex	0.423	0.391-0.451	0.454	0.424-0.527	0.522	0.426-0.615	<0.001†, 0.096‡, 0.056§
Right insular cortex	0.399	0.357-0.419	0.439	0.395-0.499	0.495	0.424-0.573	<0.001†, 0.048‡, 0.034§
Total entorhinal cortex	0.195	0.136-0.244	0.279	0.227-0.317	0.288	0.257-0.350	<0.001†, <0.001‡, 0.171§
Left entorhinal cortex	0.103	0.070-0.126	0.139	0.117-0.159	0.147	0.128-0.179	<0.001†, <0.001‡, 0.005§
Right entorhinal cortex	0.099	0.061-0.114	0.132	0.111-0.155	0.142	0.129-0.169	<0.001†, <0.001‡, 0.159§
Total thalamus	0.817	0.771-0.859	0.885	0.815-1.024	1.022	0.851-1.207	<0.001†, 0.042‡, 0.041§
Left thalamus	0.423	0.391-0.451	0.454	0.424-0.527	0.522	0.426-0.615	<0.001†, 0.096‡, 0.056§
Right thalamus	0.399	0.357-0.419	0.439	0.395-0.499	0.495	0.424-0.573	<0.001†, 0.048‡, 0.034§
Total caudate	0.416	0.358-0.435	0.436	0.405-0.497	0.484	0.428-0.585	<0.001†, 0.177‡, 0.026§
Left caudate	0.206	0.175-0.215	0.216	0.197-0.246	0.240	0.209-0.282	<0.001†, 0.120‡, 0.078§
Right caudate	0.208	0.182-0.225	0.221	0.201-0.248	0.245	0.220-0.302	<0.001†, 0.210‡, 0.014§
Total putamen	0.530	0.491-0.576	0.614	0.543-0.674	0.687	0.598-0.802	<0.001†, 0.003‡, 0.013§
Left putamen	0.267	0.238-0.284	0.306	0.278-0.339	0.351	0.296-0.395	<0.001†, 0.002‡, 0.028§
Right putamen	0.263	0.242-0.296	0.306	0.276-0.339	0.348	0.300-0.408	<0.001†, 0.006‡, 0.015§
Total pallidum	0.247	0.230-0.279	0.281	0.241-0.318	0.289	0.263-0.346	<0.001†, 0.049‡, 0.203§
Left pallidum cortex	0.125	0.120-0.137	0.143	0.122-0.158	0.144	0.132-0.171	<0.001†, 0.030‡, 0.052§
Right pallidum cortex	0.123	0.110-0.140	0.138	0.120-0.154	0.144	0.131-0.173	<0.001†, 0.072‡, 0.096§
Total hippocampus	0.419	0.359-0.480	0.563	0.475-0.645	0.638	0.544-0.758	<0.001†, <0.001‡, 0.051§
Left hippocampus	0.209	0.177-0.237	0.270	0.236-0.309	0.310	0.269-0.372	<0.001†, <0.001‡, 0.044§
Right hippocampus	0.199	0.175-0.252	0.291	0.231-0.334	0.327	0.274-0.379	<0.001†, <0.001‡, 0.067§
Total amygdala	0.157	0.124-0.182	0.218	0.186-0.244	0.253	0.218-0.303	<0.001†, <0.001‡, 0.017§
Left amygdala	0.071	0.060-0.084	0.102	0.092-0.119	0.120	0.103-0.142	<0.001†, <0.001‡, 0.037§
Right amygdala	0.088	0.066-0.098	0.113	0.096-0.133	0.135	0.115-0.159	<0.001†, <0.001‡, 0.009§
Total nucleus accumbens	0.041	0.032-0.048	0.055	0.047-0.064	0.065	0.054-0.082	<0.001†, <0.001‡, 0.039§
Left nucleus accumbens	0.017	0.013-0.023	0.025	0.021-0.031	0.030	0.025-0.039	<0.001†, 0.001‡, 0.025§
Right nucleus accumbens	0.024	0.019-0.027	0.031	0.026-0.037	0.035	0.028-0.045	<0.001†, <0.001‡, 0.062§

Data are presented as median and IQR or interquartile range (%). P-value corresponds to Independent-Samples Kruskal-Wallis Test. Pairwise comparisons of each group: † = Dementia vs NC p-value, ‡ = Dementia vs MCI p-value and § = MCI vs NC p-value. MCI, Mild cognitive impairment; NC, Normal control

Table S1.3. Cortical thickness

Cortical thickness	Dementia (n=30)		MCI (n=38)		NC (n=48)		p-value
	Median	IQR	Median	IQR	Median	IQR	
Total frontal cortex	4.692	4.502-4.843	4.820	4.661-4.911	4.876	4.670-5.031	<0.004†, 0.093‡, 0.937§
Left frontal cortex	2.357	2.265-2.425	2.408	2.346-2.478	2.449	2.324-2.532	0.004†, 0.065‡, 1.000§
Right frontal cortex	2.336	2.223-2.414	2.384	2.323-2.440	2.414	2.324-2.508	0.009†, 0.227‡, 0.705§
Total parietal cortex	4.122	3.898-4.296	4.248	4.134-4.411	4.314	4.151-4.439	0.001†, 0.021‡, 1.000§
Left parietal cortex	2.085	1.952-2.148	2.141	2.055-2.219	2.153	2.091-2.221	0.003†, 0.041‡, 1.000§
Right parietal cortex	2.036	1.923-2.137	2.115	2.067-2.198	2.138	2.080-2.218	<0.001†, 0.019‡, 0.891§
Total temporal cortex	5.059	4.765-5.267	5.395	5.261-5.545	5.556	5.419-5.695	<0.001†, 0.002‡, 0.096§
Left temporal cortex	2.506	2.361-2.619	2.702	2.631-2.772	2.776	2.659-2.850	<0.001†, 0.001‡, 0.354§
Right temporal cortex	2.502	2.410-2.643	2.715	2.616-2.780	2.798	2.702-2.851	<0.001†, 0.004‡, 0.036§
Total occipital cortex	3.687	3.592-3.794	3.846	3.709-3.949	3.855	3.735-3.936	0.001†, 0.002‡, 1.000§
Left occipital cortex	1.842	1.770-1.909	1.910	1.843-1.954	1.902	1.857-1.947	0.009†, 0.007‡, 1.000§
Right occipital cortex	1.847	1.804-1.910	1.943	1.872-2.002	1.947	1.874-2.011	<0.001†, 0.002‡, 1.000§
Total cingulate cortex	4.769	4.521-4.972	4.985	4.771-5.080	5.053	4.762-5.225	0.002†, 0.075‡, 0.741§
Left cingulate cortex	2.414	2.287-2.478	2.510	2.397-2.579	2.550	2.391-2.653	0.002†, 0.058‡, 1.000§
Right cingulate cortex	2.363	2.287-2.513	2.481	2.371-2.533	2.510	2.389-2.589	0.032†, 0.557‡, 0.641§
Total Insular cortex	5.150	4.887-5.666	5.755	5.502-6.005	5.785	5.592-6.026	<0.001†, <0.001‡, 1.000§
Left insular cortex	2.646	2.429-2.861	2.900	2.795-3.009	2.893	2.758-3.075	<0.001†, 0.001‡, 1.000§
Right insular cortex	2.555	2.427-2.765	2.866	2.698-2.987	2.894	2.725-3.017	<0.001†, <0.001‡, 1.000§
Total entorhinal cortex	5.859	4.485-6.697	7.116	6.179-7.471	7.302	6.954-7.601	<0.001†, 0.001‡, 0.280§
Left entorhinal cortex	2.944	2.152-3.421	3.570	3.087-3.747	3.630	3.465-3.801	<0.001†, 0.001‡, 0.507§
Left occipital cortex	2.950	2.248-3.367	3.578	3.137-3.820	3.663	3.451-3.905	<0.001†, 0.002‡, 0.437§

Data are presented as median and IQR or interquartile range (mm). P-value corresponds to Independent-Samples Kruskal-Wallis Test. Pairwise comparisons of each group: † = Dementia vs NC p-value, ‡ = Dementia vs MCI p-value and § = MCI vs NC p-value. MCI, Mild cognitive impairment; NC, Normal control

Supplementary 2

Table S2.1. ROC curve analysis: dementia (n = 30) vs non-dementia (n = 86)

Segmental brain volume	AUC	p-value	Cut-off (ml)	Sens	Spec	PLR	NLR	PPV	NPV	ACC
Right cerebral cortex	0.805	<0.001	185.417	80.00	74.40	3.13	0.27	52.17	91.43	75.86
Total temporal cortex	0.823	<0.001	85.335	80.00	76.70	3.44	0.26	54.55	91.67	77.59
Left temporal cortex	0.815	<0.001	42.778	70.00	80.20	3.54	0.37	55.26	88.46	77.59
Right temporal cortex	0.810	<0.001	42.395	70.00	70.90	2.41	0.42	45.65	87.14	70.69
Total parietal cortex	0.801	<0.001	86.300	70.00	75.60	2.87	0.40	50.00	87.84	74.14
Rigth parietal cortex	0.831	<0.001	42.458	70.00	83.70	4.30	0.36	60.00	88.89	80.17
Total entorhinal cortex	0.821	<0.001	3.263	83.30	70.90	2.87	0.23	50.00	92.42	74.14
Right entorhinal cortex	0.809	<0.001	1.600	76.70	72.10	2.75	0.32	48.94	89.86	73.28
Total hippocampus	0.910	<0.001	6.883	86.70	86.00	6.21	0.15	68.42	94.87	86.21
Left hippocampus	0.897	<0.001	3.413	80.00	81.40	4.30	0.25	60.00	92.11	81.03
Right hippocampus	0.903	<0.001	3.542	86.70	83.70	5.32	0.16	65.00	94.74	84.48
Total amygdala	0.891	<0.001	2.651	83.30	87.20	6.52	0.19	69.44	93.75	86.21
Left amygdala	0.912	<0.001	1.280	86.70	81.40	4.66	0.16	61.90	94.59	82.76
Right amygdala	0.845	<0.001	1.444	80.00	76.70	3.44	0.26	54.55	91.67	77.59
Total nucleus accumbens	0.834	<0.001	0.639	70.00	86.00	5.02	0.35	63.64	86.16	81.90
Left nucleus accumbens	0.812	<0.001	0.296	73.30	74.40	2.87	0.36	50.00	88.89	74.14
Right nucleus accumbens	0.824	<0.001	0.365	70.00	76.70	3.01	0.39	51.22	88.00	75.00
**Segmental brain volume/ICV**	**AUC**	**p-value**	**Cut-off (%)**	**Sens**	**Spec**	**PLR**	**NLR**	**PPV**	**NPV**	**ACC**
Total brain/ICV	0.845	<0.001	69.430	86.70	70.90	2.98	0.19	50.96	93.85	75.00
Total ventricle/ICV	0.823	<0.001	2.265	76.67	73.30	2.87	0.32	50.00	90.00	74.14
Total cerebral cortex/ICV	0.884	<0.001	26.787	86.70	82.56	4.97	0.16	63.41	94.67	83.62
Left cerebral cortex/ICV	0.887	<0.001	13.380	83.30	82.60	4.78	0.20	62.50	93.42	82.76
Right cerebral cortex/ICV	0.882	<0.001	13.397	83.30	81.40	4.48	0.20	60.98	93.33	81.90
Total cerebral gray matter/ICV	0.866	<0.001	36.285	80.00	84.90	5.29	0.24	64.86	92.41	83.62
Subcortical cerebral gray matter/ICV	0.837	<0.001	3.530	83.30	73.30	3.12	0.23	52.08	92.65	75.86
Total frontal cortex/ICV	0.833	<0.001	9.777	80.00	70.90	2.75	0.28	48.98	91.04	73.28
Left frontal cortex/ICV	0.831	<0.001	4.842	80.00	80.20	4.05	0.25	58.54	92.00	80.17
Right frontal cortex/ICV	0.835	<0.001	4.863	80.00	72.10	2.87	0.28	50.00	91.18	74.14
Total parietal cortex/ICV	0.869	<0.001	6.284	83.30	80.20	4.22	0.21	59.52	93.24	81.03
Left parietal cortex/ICV	0.847	<0.001	3.204	86.70	73.30	3.24	0.18	53.06	94.03	76.72
Right parietal cortex/ICV	0.881	<0.001	3.120	83.30	82.60	4.78	0.20	62.50	93.42	82.76
Total temporal cortex/ICV	0.909	<0.001	6.367	86.70	81.40	4.66	0.16	61.90	94.59	82.76
Left temporal cortex/ICV	0.912	<0.001	3.182	86.70	82.60	4.97	0.16	63.41	94.67	83.62
Right temporal cortex/ICV	0.897	<0.001	3.169	83.30	80.20	4.22	0.21	59.52	93.24	81.03
Total entorhinal cortex/ICV	0.855	<0.001	0.248	80.00	77.90	3.44	0.26	54.55	91.67	77.59
Left entorhinal cortex/ICV	0.822	<0.001	0.125	76.70	73.30	2.87	0.32	50.00	90.00	74.14
Right entorhinal cortex/ICV	0.852	<0.001	0.115	83.30	81.40	4.48	0.20	60.98	93.33	81.90
Total cingulate cortex/ICV	0.871	<0.001	1.112	83.30	83.70	5.12	0.20	64.10	93.51	83.62
Left cingulate cortex/ICV	0.842	<0.001	0.589	80.00	72.10	2.87	0.28	50.00	91.18	74.14
Right cingulate cortex/ICV	0.840	<0.001	0.560	76.70	80.20	3.88	0.29	57.50	90.79	79.31
Total putamen cortex/ICV	0.835	<0.001	0.570	76.70	76.70	3.15	0.35	52.38	89.19	75.86
Left putamen/ICV	0.836	<0.001	0.280	73.30	83.70	4.50	0.32	61.11	90.00	81.03
Right putamen/ICV	0.818	<0.001	0.297	80.00	70.90	2.75	0.28	48.98	91.04	73.28
Total hippocampus/ICV	0.893	<0.001	0.486	80.00	81.40	4.30	0.25	60.00	92.11	81.03
Left hippocampus/ICV	0.889	<0.001	0.239	80.00	82.60	4.59	0.24	61.54	92.21	81.90
Right hippocampus/ICV	0.883	<0.001	0.267	93.30	75.60	3.82	0.09	57.14	97.01	80.17
total amygdala/ICV	0.914	<0.001	0.185	80.00	86.00	5.73	0.23	66.67	92.50	84.48
Left amygdala/ICV	0.933	<0.001	0.092	86.70	87.20	6.78	0.15	70.27	94.94	87.07
Right amygdala/ICV	0.878	<0.001	0.101	80.00	82.60	4.59	0.24	61.54	92.21	81.90
Total nucleus accumbens/ICV	0.865	<0.001	0.051	80.00	74.40	3.13	0.27	52.17	91.43	75.86
Left nucleus accumbens/ICV	0.854	<0.001	0.023	80.00	74.40	3.13	0.27	52.17	91.43	75.86
Right nucleus accumbens/ICV	0.855	<0.001	0.027	76.70	73.30	2.87	0.32	50.00	90.00	74.14
**Thickness**	**AUC**	**p-value**	**Cut-off (mm)**	**Sens**	**Spec**	**PLR**	**NLR**	**PPV**	**NPV**	**ACC**
Total temporal cortex	0.816	<0.001	5.342	80.00	74.40	3.13	0.27	52.17	91.43	75.86
Left temporal cortex	0.817	<0.001	2.655	80.00	75.60	3.28	0.26	53.33	91.55	76.72
Right temporal cortex	0.814	<0.001	2.660	80.00	74.40	3.13	0.27	52.17	91.43	75.86
Total entorhinal cortex	0.807	<0.001	6.870	83.30	72.10	2.88	0.24	51.02	92.54	75.00
Left entorhinal cortex	0.800	<0.001	3.225	73.30	80.20	3.71	0.33	56.41	89.61	78.45
Right insular cortex	0.806	<0.001	2.695	73.30	80.20	3.71	0.33	56.41	89.61	78.45

This table presents only parameters that have AUC ≥ 0.8.
ACC, accuracy; AUC, area under the curve; NLR, negative likelihood ratio; NPV, negative predictive value; PLR, positive likelihood ratio; PPV, positive predictive value; sens, sensitivity; spec, specificity

Table S2.2. ROC curve analysis: dementia (n = 30) vs NC (n = 48)

**Segmental brain volume**	**AUC**	**p-value**	**Cut-off (ml)**	**Sens**	**Spec**	**PLR**	**NLR**	**PPV**	**NPV**	**ACC**
Total ventricle	0.830	<0.001	24.816	86.70	75.00	3.47	0.18	68.42	90.00	79.49
Total ventricle/ICV	0.847	<0.001	2.074	83.30	75.00	3.33	0.22	67.57	87.80	78.21
Total cerebral cortex	0.846	<0.001	378.327	86.70	72.90	3.20	0.18	66.67	89.74	78.21
Left cerebral cortex	0.835	<0.001	185.298	76.70	77.10	3.35	0.30	67.65	84.09	76.92
Right cerebral cortex	0.853	<0.001	189.337	86.70	72.90	3.20	0.18	66.67	89.74	78.21
Total cerebral gray matter	0.832	<0.001	521.189	76.70	72.90	2.83	0.32	63.89	83.33	74.36
Subcortical cerebral gray matter	0.862	<0.001	49.590	83.30	70.80	2.86	0.24	64.10	87.18	75.64
Total parietal cortex	0.849	<0.001	87.384	76.70	77.10	3.35	0.30	67.65	84.09	76.92
Left parietal cortex	0.807	<0.001	44.021	73.30	70.80	2.51	0.38	61.11	80.95	71.79
Right parietal cortex	0.874	<0.001	44.907	86.70	72.90	3.20	0.18	66.67	89.74	78.21
Total temporal cortex	0.878	<0.001	85.335	80.00	83.30	4.80	0.24	75.00	86.96	82.05
Left temporal cortex	0.876	<0.001	45.309	86.70	75.00	3.47	0.18	68.42	90.00	79.49
Right temporal cortex	0.856	<0.001	43.433	83.30	72.90	3.08	0.23	65.79	87.50	76.92
Total entorhinal cortex	0.856	<0.001	3.263	83.30	83.30	5.00	0.20	75.76	88.89	83.33
Right entorhinal cortex	0.845	<0.001	1.633	80.00	72.90	3.84	0.25	70.59	86.36	79.49
Total putamen	0.848	<0.001	8.107	80.00	77.10	3.49	0.26	68.57	86.05	78.21
Left putamen	0.806	<0.001	3.972	73.30	72.90	3.52	0.34	68.75	82.61	76.92
Right putamen	0.862	<0.001	4.056	83.30	81.20	4.44	0.21	73.53	88.64	82.05
Total hippocampus	0.953	<0.001	7.209	90.00	85.40	6.17	0.12	79.41	93.18	87.18
Left hippocampus	0.944	<0.001	3.520	93.30	83.30	5.60	0.08	77.78	95.24	87.18
Right hippocampus	0.941	<0.001	3.678	93.30	83.30	5.60	0.08	77.78	95.24	87.18
Total amygdala	0.939	<0.001	2.827	86.70	87.50	6.93	0.15	81.25	91.30	87.18
Left amygdala	0.951	<0.001	1.349	90.00	87.50	7.20	0.11	81.82	93.33	88.46
Right amygdala	0.908	<0.001	1.445	80.00	89.60	7.68	0.22	82.76	87.76	85.90
Total nucleus accumbens	0.867	<0.001	0.688	76.70	79.20	3.68	0.29	69.70	84.44	78.21
Left nucleus accumbens	0.849	<0.001	0.291	70.00	89.60	6.72	0.33	80.77	82.69	82.05
Right nucleus accumbens	0.859	<0.001	0.394	80.00	72.90	2.95	0.27	64.86	85.37	75.64
**Segmental brain volume/ICV**	**AUC**	**p-value**	**Cut-off (%)**	**Sens**	**Spec**	**PLR**	**NLR**	**PPV**	**NPV**	**ACC**
Total brain/ICV	0.901	<0.001	70.860	90.00	81.20	4.80	0.12	75.00	92.86	84.62
Total cerebral cortex/ICV	0.923	<0.001	26.902	86.67	87.50	6.93	0.15	81.25	91.30	87.18
Left cerebral cortex/ICV	0.923	<0.001	14.053	90.00	81.20	4.80	0.12	75.00	92.86	84.62
Right cerebral cortex/ICV	0.926	<0.001	14.108	90.00	81.20	4.80	0.12	75.00	92.86	84.62
Total cerebral white matter/ICV	0.831	<0.001	30.880	86.70	72.90	3.20	0.18	66.67	89.74	78.21
Left cerebral white matter/ICV	0.832	<0.001	15.596	90.00	72.90	3.32	0.14	67.50	92.11	79.49
Right cerebral white matter/ICV	0.821	<0.001	15.405	80.00	72.90	2.95	0.27	64.86	85.37	75.64
Total cerebral gray matter/ICV	0.915	<0.001	36.971	83.30	85.40	5.71	0.20	78.12	89.13	84.62
Subcortical cerebral gray matter/ICV	0.889	<0.001	3.541	86.70	83.30	5.20	0.16	76.47	90.91	84.62
Total frontal cortex/ICV	0.880	<0.001	9.777	80.00	83.30	4.80	0.24	75.00	86.96	82.05
Left frontal cortex/ICV	0.875	<0.001	4.847	80.00	85.40	5.49	0.23	77.42	87.23	83.33
Right frontal cortex/ICV	0.884	<0.001	4.946	83.30	81.20	4.44	0.21	73.53	88.64	82.05
Total parietal cortex/ICV	0.915	<0.001	6.383	86.70	89.60	8.32	0.15	83.87	91.49	88.46
Left parietal cortex/ICV	0.899	<0.001	3.205	86.70	83.30	5.20	0.16	76.47	90.91	84.62
Rigth parietal cortex/ICV	0.925	<0.001	3.222	86.70	89.60	8.32	0.15	83.87	91.49	88.46
Total temporal cortex/ICV	0.945	<0.001	6.514	93.30	83.30	5.60	0.08	77.78	95.24	87.18
Left temporal cortex/ICV	0.951	<0.001	3.264	90.00	85.40	6.17	0.12	79.41	93.18	87.18
Right temporal cortex/ICV	0.931	<0.001	3.214	86.70	85.40	5.94	0.16	78.79	91.11	85.90
Total entorhinal cortex/ICV	0.896	<0.001	0.248	80.00	89.60	7.68	0.22	82.76	87.76	85.90
Left entorhinal cortex/ICV	0.855	<0.001	0.132	80.00	70.80	2.74	0.28	63.16	85.00	74.36
Right entorhinal cortex/ICV	0.894	<0.001	0.128	86.70	81.20	4.62	0.16	74.29	90.47	83.33
Total occipital cortex/ICV	0.824	<0.001	2.933	80.00	72.90	2.95	0.27	64.86	85.37	75.64
Left occipital cortex/ICV	0.821	<0.001	1.403	73.30	72.90	2.71	0.37	62.86	81.40	73.08
Right occipital cortex/ICV	0.831	<0.001	1.542	80.00	70.80	2.74	0.28	63.16	85.00	74.36
Total cingulate cortex/ICV	0.913	<0.001	1.167	90.00	85.40	6.17	0.12	79.41	93.18	87.18
Left cingulate cortex/ICV	0.917	<0.001	0.605	90.00	79.20	4.32	0.13	72.97	92.68	83.33
Right cingulate cortex/ICV	0.872	<0.001	0.576	80.00	75.00	3.20	0.27	66.67	85.71	76.92
Total Thalamus/ICV	0.810	<0.001	0.883	83.30	75.00	3.33	0.22	67.57	87.80	78.21
Right thalamus/ICV	0.822	<0.001	0.429	83.30	75.00	3.33	0.22	67.57	87.80	78.21
Total Putamen/ICV	0.899	<0.001	0.596	86.70	77.10	3.78	0.17	70.27	90.24	80.77
Left putamen/ICV	0.890	<0.001	0.297	86.70	75.00	3.47	0.18	68.42	90.00	79.49
Right putamen/ICV	0.881	<0.001	0.297	80.00	79.20	3.84	0.25	70.59	86.36	79.49
Total hippocampus/ICV	0.929	<0.001	0.486	80.00	91.70	9.60	0.22	85.71	88.00	87.18
Left hippocampus/ICV	0.924	<0.001	0.252	86.70	83.30	5.20	0.16	76.47	90.91	84.62
Right hippocampus/ICV	0.919	<0.001	0.267	93.30	81.20	4.98	0.08	75.68	95.12	85.90
Total amygdala/ICV	0.956	<0.001	0.216	96.70	81.20	5.16	0.04	76.32	97.50	87.18
Left amygdala/ICV	0.964	<0.001	0.099	96.70	83.30	5.80	0.04	78.38	97.56	88.46
Right amygdala/ICV	0.932	<0.001	0.110	86.70	87.50	6.93	0.15	81.25	91.30	87.18
Total nucleus accumbens/ICV	0.906	<0.001	0.052	86.70	81.20	4.62	0.16	74.29	90.70	83.33
Left nucleus accumbens/ICV	0.893	<0.001	0.023	80.00	81.20	4.27	0.25	72.73	86.67	80.77
Right nucleus accumbens/ICV	0.901	<0.001	0.028	86.70	75.00	3.47	0.18	68.42	90.00	79.49
**Thickness**	**AUC**	**p-value**	**Cut-off (mm)**	**Sens**	**Spec**	**PLR**	**NLR**	**PPV**	**NPV**	**ACC**
Total temporal cortex	0.847	<0.001	5.409	86.70	79.20	4.16	0.17	72.22	90.48	82.05
Left temporal cortex	0.837	<0.001	2.655	80.00	77.10	3.49	0.26	68.57	86.05	78.21
Right temporal cortex	0.850	<0.001	2.660	80.00	81.20	4.27	0.25	72.73	86.67	80.77
Total entorhinal cortex	0.842	<0.001	6.879	83.30	79.20	4.00	0.21	71.43	88.37	80.77
Left entorhinal cortex	0.831	<0.001	3.490	83.30	72.90	3.08	0.23	65.79	87.50	76.92
Right entorhinal cortex	0.832	<0.001	3.383	80.00	83.30	4.80	0.24	75.00	86.96	82.05
Right insular cortex	0.815	<0.001	2.701	73.30	81.20	3.91	0.33	70.97	82.98	78.21

This table presents only parameters that have AUC ≥ 0.8.

ACC, accuracy; AUC, area under the curve; NLR, negative likelihood ratio; NPV, negative predictive value; PLR, positive likelihood ratio; PPV, positive predictive value; sens, sensitivity; spec, specificity

Table S2.3. ROC curve analysis: dementia (n = 30) vs MCI (n = 38)

**Segmental brain volume**	**AUC**	**p-value**	**Cut-off (ml)**	**Sens**	**Spec**	**PLR**	**NLR**	**PPV**	**NPV**	**ACC**
Total hippocampus	0.856	<0.001	6.724	80.00	78.90	3.80	0.25	75.00	83.33	79.41
Left hippocampus	0.838	<0.001	3.363	76.70	71.10	2.65	0.33	67.65	79.41	73.53
Right hippocampus	0.856	<0.001	3.396	80.00	81.60	4.34	0.25	77.42	83.78	80.88
Total amygdala	0.831	<0.001	2.651	83.30	76.30	3.52	0.22	73.53	88.64	82.05
Left amygdala	0.861	<0.001	1.181	80.00	86.80	6.08	0.23	82.76	84.62	83.82
**Segmental brain volume/ICV**	**AUC**	**p-value**	**Cut-off (%)**	**Sens**	**Spec**	**PLR**	**NLR**	**PPV**	**NPV**	**ACC**
Total cerebral cortex/ICV	0.836	<0.001	26.787	86.67	76.32	3.66	0.17	74.29	87.88	80.88
Left cerebral cortex/ICV	0.841	<0.001	13.343	80.00	76.30	3.38	0.26	72.73	82.86	77.94
Right cerebral cortex/ICV	0.826	<0.001	13.397	83.30	73.70	3.17	0.23	71.43	84.85	77.94
Total cerebral gray matter/ICV	0.805	<0.001	36.330	80.00	78.90	3.80	0.25	75.00	83.33	79.41
Total temporal cortex/ICV	0.864	<0.001	6.367	86.70	73.70	3.29	0.18	72.22	87.50	79.41
Left temporal cortex/ICV	0.864	<0.001	3.135	80.00	76.30	3.38	0.26	72.73	82.86	77.94
Right temporal cortex/ICV	0.853	<0.001	3.169	83.30	73.70	3.17	0.23	71.43	84.85	77.94
Total parietal/ICV	0.812	<0.001	6.244	80.00	71.10	2.76	0.28	68.57	81.82	75.00
Right parietal cortex/ICV	0.826	<0.001	3.120	83.30	71.10	2.88	0.23	69.44	84.38	76.47
Total cingulate cortex/ICV	0.818	<0.001	1.105	80.00	78.90	3.80	0.25	75.00	83.33	79.41
Total entorhinal cortex/ICV	0.804	<0.001	0.232	70.00	73.70	2.66	0.41	67.74	75.68	72.06
Total hippocampus/ICV	0.848	<0.001	0.479	76.70	76.30	3.24	0.31	71.88	80.56	76.47
Left hippocampus/ICV	0.846	<0.001	0.239	80.00	73.70	3.04	0.27	70.59	82.35	76.47
Right hippocampus/ICV	0.838	<0.001	0.241	70.00	73.70	2.66	0.41	67.74	75.68	72.06
Total amygdala/ICV	0.861	<0.001	0.186	80.00	76.30	3.38	0.26	72.73	82.86	77.94
Left amygdala/ICV	0.893	<0.001	0.089	83.30	84.20	5.28	0.20	80.65	86.49	83.82
Right amygdala/ICV	0.810	<0.001	0.101	80.00	71.10	2.76	0.28	68.57	81.82	75.00
Total nucleus accumbens/ICV	0.814	<0.001	0.048	76.70	76.30	3.24	0.31	71.88	80.56	76.47
Left nucleus accumbens/ICV	0.805	<0.001	0.022	73.30	73.70	2.79	0.36	68.75	77.78	75.53

This table presents only parameters that have AUC ≥ 0.8.
ACC, accuracy; AUC, area under the curve; NLR, negative likelihood ratio; NPV, negative predictive value; PLR, positive likelihood ratio; PPV, positive predictive value; sens, sensitivity; spec, specificity

Table S2.4. ROC curve analysis: MCI (n = 38) vs NC (n = 48)

Segmental brain volume	AUC	p-value	Cut-off (ml)	Sens	Spec	PLR	NLR	PPV	NPV	ACC
Total hippocampus	0.856	<0.001	6.724	80.00	78.90	3.80	0.25	75.00	83.33	79.41
Left hippocampus	0.838	<0.001	3.363	76.70	71.10	2.65	0.33	67.65	79.41	73.53
Right hippocampus	0.856	<0.001	3.396	80.00	81.60	4.34	0.25	77.42	83.78	80.88
Total amygdala	0.831	<0.001	2.651	83.30	76.30	3.52	0.22	73.53	88.64	82.05
Left amygdala	0.861	<0.001	1.181	80.00	86.80	6.08	0.23	82.76	84.62	83.82
**Segmental brain volume/ICV**	**AUC**	**p-value**	**Cut-off (%)**	**Sens**	**Spec**	**PLR**	**NLR**	**PPV**	**NPV**	**ACC**
Total cerebral cortex/ICV	0.836	<0.001	26.787	86.67	76.32	3.66	0.17	74.29	87.88	80.88
Left cerebral cortex/ICV	0.841	<0.001	13.343	80.00	76.30	3.38	0.26	72.73	82.86	77.94
Right cerebral cortex/ICV	0.826	<0.001	13.397	83.30	73.70	3.17	0.23	71.43	84.85	77.94
Total cerebral gray matter/ICV	0.805	<0.001	36.330	80.00	78.90	3.80	0.25	75.00	83.33	79.41
Total temporal cortex/ICV	0.864	<0.001	6.367	86.70	73.70	3.29	0.18	72.22	87.50	79.41
Left temporal cortex/ICV	0.864	<0.001	3.135	80.00	76.30	3.38	0.26	72.73	82.86	77.94
Right temporal cortex/ICV	0.853	<0.001	3.169	83.30	73.70	3.17	0.23	71.43	84.85	77.94
Total parietal/ICV	0.812	<0.001	6.244	80.00	71.10	2.76	0.28	68.57	81.82	75.00
Right parietal cortex/ICV	0.826	<0.001	3.120	83.30	71.10	2.88	0.23	69.44	84.38	76.47
Total cingulate cortex/ICV	0.818	<0.001	1.105	80.00	78.90	3.80	0.25	75.00	83.33	79.41
Total entorhinal cortex/ICV	0.804	<0.001	0.232	70.00	73.70	2.66	0.41	67.74	75.68	72.06
Total hippocampus/ICV	0.848	<0.001	0.479	76.70	76.30	3.24	0.31	71.88	80.56	76.47
Left hippocampus/ICV	0.846	<0.001	0.239	80.00	73.70	3.04	0.27	70.59	82.35	76.47
Right hippocampus/ICV	0.838	<0.001	0.241	70.00	73.70	2.66	0.41	67.74	75.68	72.06
Total amygdala/ICV	0.861	<0.001	0.186	80.00	76.30	3.38	0.26	72.73	82.86	77.94
Left amygdala/ICV	0.893	<0.001	0.089	83.30	84.20	5.28	0.20	80.65	86.49	83.82
Right amygdala/ICV	0.810	<0.001	0.101	80.00	71.10	2.76	0.28	68.57	81.82	75.00
Total nucleus accumbens/ICV	0.814	<0.001	0.048	76.70	76.30	3.24	0.31	71.88	80.56	76.47
Left nucleus accumbens/ICV	0.805	<0.001	0.022	73.30	73.70	2.79	0.36	68.75	77.78	75.53

This table presents only parameters that have AUC ≥ 0.8.
ACC, accuracy; AUC, area under the curve; NLR, negative likelihood ratio; NPV, negative predictive value; PLR, positive likelihood ratio; PPV, positive predictive value; sens, sensitivity; spec, specificity

Supplementary 3

Table S3. Linear regression analysis.

Brain parameters	Dementia vs non-dementia	Dementia vs NC	Dementia vs MCI	MCI vs NC
Total brain volume (ml)	-63.316 (-96.526, -30.106)*	-90.918 (-134.807, -47.029)*	-62.370 (-97.010, -27.730)*	-15.726 (-47.702, 16.250)
Total ventricle volume (ml)	11.324 (5.552, 17.097)*	12.314 (4.288, 20.340)*	11.027 (4.375, 17.679)*	-1.700 (-6.719, 3.319)
Total cerebral cortex volume (ml)	-40.079 (-54.415, -25.742)*	-53.659 (-72.953, -34.366)*	-39.211 (-54.150, -24.271)*	-10.433 (-23.152, 2.258)
Total hippocampal volume (ml)	-13.734 (-17.505, -9.963)*	-14.951 (-19.670, -10.232)*	-13.426 (-18.255, -8.597)*	-1.382 (-4.589, 1.826)
Left hippocampus volume (ml)	-0.632 (-0.813, -0.451)*	-0.701 (-0.937, -0.466)*	-0.626 (-0.856, -0.397)*	-0.067 (-0.216, 0.082)
Right hippocampus volume (ml)	-0.742 (-0.956, -0.527)*	-0.794 (-1.059, -0.529)*	-0.716 (-0.990, -0.397)*	-0.071 (-0.257, 0.115)
Total amygdala volume (ml)	-7.195 (-9.277, -5.113)*	-9.012 (-11.699, -6.326)*	-6.557 (-9.072, -4.042)*	-1.855 (-3.694, -0.077)*
Left amygdala volume (ml)	-0.394 (-0.493, -0.296)*	-0.474 (-0.603, -0.344)*	-0.363 (-0.480, -0.246)*	-0.090 (-0.177, -0.003)*
Right amygdala volume (ml)	-0.325 (-0.446, -0.205)*	-0.428 (-0.580, -0.275)*	-0.293 (-0.441, -0.145)*	-0.099 (-0.205, 0.0047)
Total brain volume/ICV (%)	-8.693 (-13.869, -3.528)*	-13.575 (-20.184, -6.966)*	-7.342 (-12.110, -2.574)*	-2.615 (-8.230, 3.000)
Total ventricle/ICV (%)	0.999 (0.563, 1.436)*	1.328 (0.728, 1.928)*	0.951 (0.464, 1.438)*	-0.042 (-0.436, 0.352)
Total cerebral cortex /ICV (%)	-4.399 (-6.458, -2.339)*	-6.525 (-9.231, -3.818)*	-3.834 (-5.710, -1.958)*	-0.696 (-1.758, 0.366)
Total hippocampus/ICV (%)	-0.137 (-0.189, -0.086)*	-0.175 (-0.242, -0.109)*	-0.124 (-0.177, -0.071)*	-0.026 (-0.078, 0.026)
Left hippocampus/ICV (%)	-0.064 (-0.089, -0.040)*	-0.083 (-0.115, -0.051)*	-0.058 (-0.083, -0.033)*	-0.013 (-0.038, 0.012)
Right hippocampus volume/ICV (%)	-0.073 (-0.101, -0.046)*	-0.092 (-0.128, -0.057)*	-0.066 (-0.095, -0.037)*	-0.013 (-0.041, 0.015)
Total amygdala/ICV (%)	-0.068 (-0.090, -0.045)*	-0.092 (-0.121, -0.063)*	-0.058 (-0.080, -0.035)*	-0.021 (-0.044, 0.002)
Left amygdala volume/ICV (%)	-0.037 (-0.047, -0.026)*	-0.048 (-0.062, -0.034)*	-0.032 (-0.042, -0.021)*	-0.010 (-0.021, 0.001)
Right amygdala volume/ICV (%)	-0.031 (-0.043, -0.019)*	-0.044 (-0.060, -0.029)*	-0.026 (-0.039, -0.013)*	-0.011 (-0.023, 0.002)
Total frontal cortex thickness (mm)	-0.118 (-0.234, -0.003)*	-0.109 (-0.272, 0.054)	-0.097 (-0.203, 0.009)	-0.066 (-0.187, 0.054)
Total parietal cortex thickness (mm)	-0.194 (-0.312, -0.075)*	-0.184 (-0.346, -0.022)*	-0.187 (-0.322, -0.053)*	-0.026 (-0.135, 0.083)
Total temporal cortex thickness (mm)	-0.372 (-0.551, -0.192)*	-0.419 (-0.672, -0.166)*	-0.310 (-0.502, -0.117)*	-0.128 (-0.295, 0.038)
Total occipital cortex thickness (mm)	-0.108 (-0.199, -0.016)*	-0.068 (-0.189, 0.053)	-0.129 (-0.232, -0.026)*	0.042 (-0.047, 0.131)
Total cingulate cortex thickness (mm)	-0.161 (-0.301, -0.022)*	-0.131 (-0.323, 0.061)	-0.125 (-0.270, 0.020)	-0.119 (-0.254, 0.017)
Total insular cortex thickness (mm)	-0.423 (-0.630, -0.215)*	-0.388 (-0.676, -0.101)*	-0.399 (-0.613, -0.185)*	-0.005 (-0.211, 0.201)
Total entorhinal cortex thickness(mm)	-1.553 (-1.644, -0.662)*	-1.448 (-2.100, -0.796)*	-0.942 (-1.506, -0.379)*	-0.380 (-0.831, 0.071)

* $P < 0.05$.
